# High-Resolution Profiling of Innate Immune Responses by Porcine Dendritic Cell Subsets *in vitro* and *in vivo*

**DOI:** 10.3389/fimmu.2020.01429

**Published:** 2020-07-07

**Authors:** Gaël Auray, Stephanie C. Talker, Irene Keller, Sylvie Python, Markus Gerber, Matthias Liniger, Llilianne Ganges, Rémy Bruggmann, Nicolas Ruggli, Artur Summerfield

**Affiliations:** ^1^Institute of Virology and Immunology, Mittelhäusern, Switzerland; ^2^Department of Infectious Diseases and Pathobiology, University of Bern, Bern, Switzerland; ^3^Department for Biomedical Research and Swiss Institute of Bioinformatics, University of Bern, Bern, Switzerland; ^4^OIE Reference Laboratory for Classical Swine Fever, IRTA-CReSA, Barcelona, Spain; ^5^Interfaculty Bioinformatics Unit and Swiss Institute of Bioinformatics, University of Bern, Bern, Switzerland

**Keywords:** dendritic cells, toll like receptor, transcriptomics analysis, porcine (pig) model, classical swine fever

## Abstract

The present study investigated the transcriptomic response of porcine dendritic cells (DC) to innate stimulation *in vitro* and *in vivo*. The aim was to identify DC subset-specialization, suitable Toll-like receptor (TLR) ligands targeting plasmacytoid DC (pDC), and the DC activation profile during highly and low virulent classical swine fever virus (CSFV, strain Eystrup and Pinar del Rio, respectively) infection, chosen as model for a virus causing a severe immunopathology. After identification of porcine conventional DC (cDC) 1, cDC2, pDC and a monocyte-derived subset in lymphoid tissues, we characterized DC activation using transcriptomics, and focused on chemokines, interferons, cytokines, as well as on co-stimulatory and inhibitory molecules. We demonstrate that porcine pDC provide important signals for Th1 and interferon responses, with CpG triggering the strongest responses in pDC. DC isolated early after infection of pigs with either of the two CSFV strains showed prominent upregulation of *CCL5, CXCL9, CXCL10, CXCL11*, and *XCL1*, as well as of the cytokines *TNFSF13B, IL6, IL7, IL12B, IL15, IL27*. Transcription of *IL12B* and many interferon genes were mostly restricted to pDC. Interestingly, the infection was associated with a prominent induction of inhibitory and cell death receptors. When comparing low and highly virulent CSFV strains, the latter induced a stronger inflammatory and antiviral response but a weaker cell cycle response, and reduced antigen presentation functions of DC. Taken together, we provide high-resolution information on DC activation in pigs, as well as information on how DC modulation could be linked to CSFV immunopathology.

## Introduction

Dendritic cells (DC) are sentinel innate immune cells that are present in the skin and at mucosal surfaces and are specialized in the early sensing of pathogens. Antigen uptake by DC, and their activation-induced migration to secondary lymphoid organs is vital for the initiation of specific T-cell responses.

DC can be divided into functionally specialized subsets, including two conventional DC subsets (cDC1 and cDC2) and plasmacytoid (p)DC. These subsets not only differ in their ability to induce particular T-cell responses but also in their expression of pattern recognition receptors (PRRs) which sense danger signals such as pathogen- and damage-associated molecular patterns (1). Triggering of these receptors leads to activation and maturation of DC, which is associated with a shift in their expression of chemokine receptors from CCR2 and/or CCR5 to CCR7, allowing them to migrate toward lymph nodes (LN) (2). This maturation process is also associated with an upregulation of costimulatory molecules, which are required for activation of naïve T cells. Depending on the stimuli and the immunological environment, signals from activated DC also shape the adaptive immune response toward Th1, Th2, or Th17 responses.

Importantly, the DC subset-specific expression of PRRs belonging to the Toll-like receptor (TLR) and C-type lectin receptor (CLR) family can vary in a species-dependent manner. In a previous work we identified and precisely characterized different porcine DC and monocytic cell subsets circulating in blood, and described some peculiarities of the porcine immune system (3). These data showed for example that porcine pDC express the highest levels of *TLR7* and *TLR9*, as observed in mouse and human, but surprisingly also of *TLR3*, a PRR mainly found on cDC1 in both mouse and human (4, 5). Another example is the high level of *TLR9* expression in cDC1 of pigs but not humans (3). In addition to differences in PRR expression, our data also indicated functional differences in the response of porcine DC subsets and monocytes to stimulation. Following *in vitro* stimulation of several porcine DC subsets with TLR3, TLR7, or TLR9 ligands, pDC were the main producers of TNF, IFN-α, and IL-12p40 and were the only subset to express IL-12p35, suggesting pDC to be the main source of inflammatory cytokines and IL-12 in the pig. Surprisingly, pDC were also found to be responsive to a TLR2 ligand, and even to a TLR4 ligand in terms of TNF production, again an observation not found with human pDC. Furthermore, the upregulation of costimulatory molecules and CCR7 on cDC was often dependent on the presence of pDC in the cultures (3, 6). This would indicate that in the pig, pDC play a particularly pivotal role in sensing viruses through TLR3, TLR7, and TLR9 and in sensing other microbial organisms through cell surface TLR, resulting in cytokine production and in support for cDC activation.

Considering the central immunological importance of this concept, the goal of this study was to follow up on our previous work and to employ RNA-sequencing (RNA-Seq)-based transcriptomics to characterize in more detail the immune response profile of sorted blood DC subsets following *in vitro* TLR stimulation. Because of important species differences in the functional specialization of DC subsets described above, our main aim was to dissect subset-dependent differences in the expression of co-stimulatory molecules, and the expression of cytokines and chemokines as well as their receptors. To this end, we investigated the transcriptional response of cDC1, cDC2, pDC and monocytes to a TLR1/2 ligand, known to directly activate all porcine DC subsets (3). Considering the apparent important role of pDC in pathogen sensing in the pig, we additionally investigated the activation profile of pDC following stimulation with TLR3, TLR7, TLR7/8, or TLR9 ligands. In view of the fact that *in vitro* stimulation of isolated DC might not necessarily reflect the *in vivo* situation, these data were complemented with *in vivo* results obtained during classical swine fever virus (CSFV) infection. Before doing so, we characterized cDC1, cDC2, pDC and monocytic cells in lymph nodes and tonsils at homeostasis by flow cytometry and RNA-Seq. Having achieved a clear identification of the above-mentioned subsets in secondary lymphoid organs, we characterized their transcriptomic profiles following infection with the highly virulent CSFV strain Eystrup (vEy-37) (7) and the low virulent Pinar del Rio (PdR) strain (8). We chose CSFV as a model because of the extensive knowledge on CSFV interaction with the innate immune system and the strong immunopathological effect of this infection (9). In fact, infection of pigs is associated with strong innate immune responses, which includes both an inflammatory cytokine and interferon type I response that appears to be associated with a severe depletion of lymphocytes in primary and secondary lymphoid tissue (9–11). As this response is known to correlate with the virulence of the CSFV strain (12), we decided to compare a low and a highly virulent strain of CSFV. Altogether, this work represents an important step in understanding the functional specialization of DC subsets in the pig.

## Materials and Methods

### Isolation of Peripheral Blood Mononuclear Cells, and Cells From Lymph Nodes and Tonsils

For the *in vitro* stimulation experiments, blood sampling was performed on three 14- to 18-months old Large White pigs kept under specific pathogen-free (SPF) conditions at the Institute of Virology and Immunology (IVI, Mittelhäusern, Switzerland). These studies were performed under the licenses BE88/14 and BE131/17, which were reviewed by the cantonal committee on animal experiments of the canton of Bern, Switzerland, and approved by the cantonal veterinary authority (Amt für Landwirtschaft und Natur LANAT, Veterinärdienst VeD, Bern, Switzerland). PBMC were isolated by centrifugation on a Ficoll-paque density gradient (1.077 g/L, GE Healthcare. Chicago, IL, USA). For the isolation and characterization of DC subsets from lymph nodes and tonsils, mandibular and retropharyngeal lymph nodes and tonsils were obtained from SPF pigs slaughtered at the IVI (Animal facility license BE-VTH-3/14). The pigs were not slaughtered for the purpose of organ collection. Consent for collecting samples after slaughter was obtained from the animal facility manager and the animal welfare officer of the IVI. Harvested organs were washed with sterile PBS, cut in small pieces (≈ 0.3 cm^3^), and incubated in 20 mL of DMEM with Glutamax (Thermofisher, Waltham MA, USA) supplemented with 10% fetal bovine serum (FBS; Sigma-Aldrich, Buchs, Switzerland) containing 1 mg/mL collagenase D (Sigma-Aldrich) and 100 μg/mL DNase I (Sigma-Aldrich) during 15 min under agitation at 37°C. The enzymatic reaction was stopped by adding 2 mL of PBS with 5 mM EDTA solution. The cells were then filtered through a 100 μm filter (BD Biosciences, Allschwil, Switzerland), washed two times with PBS (4°C) and finally frozen in liquid nitrogen at 10^7^ cells/mL in freezing medium consisting of DMEM with 40% FBS and 10% DMSO until further use.

### Flow Cytometry and Cell Sorting

The flow cytometry panels used to define DC subsets and monocytic cells in blood and lymphoid tissues were based on our previous work defining cDC1 as CD135^+^CD14^−^CD172a^−/low^CADM1^+^wCD11R1^+^, cDC2 as CD135^+^CD14^−^CD172a^+^CADM1^+^CD115^+^wCD11R1^+^CD1^+^, pDC as CD135^+^CD14^−^CD4^+^CD172a^+^CD123^+^CD303^+^ and monocytes as CD135^−^CD14^+^CD172a^high^ (3). The basic staining protocol employed was a four-step five-color staining described previously (3). Briefly, cells were incubated with primary antibodies including anti-CD172a (clone 77-22-15A, kindly given by Dr. Armin Saalmüller, Veterinary University of Vienna, Austria) and anti-SynCAM (TSLC1/CADM1) (cross-reactive anti-mouse Syn-CAM mAb, clone 3E1, MBL International, Woburn, MA, USA), followed by a second incubation step with the corresponding secondary antibodies anti-mouse IgG2b Alexa Fluor 647 (Thermofisher) and anti-chicken IgY biotin (Jackson Immunoresearch Laboratories, West Grove, PA, USA). After a third step of Ig blocking (ChromPure mouse IgG 100 ng/ml; Jackson Immunoresearch), cells were finally incubated with directly conjugated antibodies anti-CD14-FITC (clone MIL2, Bio-Rad AbD Serotec, Oxford, UK) and anti-CD4-PerCP-Cy5.5 (clone 74-12-4, BD Biosciences), and with V500-coupled streptavidin (BD Biosciences). The cell-surface expression of several markers was determined to further characterize the DC subsets using the following antibodies: anti-wCD11R1 (clone MIL4, Bio-Rad), anti-CD1 (clone 76-7-4, kindly provided by Dr. Armin Saalmüller), anti-CD115 (CSF-1R, clone ROS8G11-1, kindly provided by Dr. David Hume, Roslin Institute, University of Edinburgh, UK), anti-CD205 (clone ZH9F7, kindly provided by Dr. Jesus Hernandez, Centro de Investigación en Alimentación y Desarrollo, Hermosillo, Mexico) (13), anti-CD207 (cross-reactive anti-mouse langerin, clone DDX0368, Dendritics, Lyon, France), anti-MHC II (SLA-DQ, clone TH16B, Washington State University Monoclonal Antibody Center, WA, USA), human CD152-mu Ig (human CTLA fusion protein recognizing CD80/86, Ancell Corporation, Stillwater, MN, USA), or His-tagged porcine recombinant protein IL-3 or Flt3L (14, 15). The secondary antibodies used were anti-mouse IgG1-RPE, anti-mouse IgG2a-RPE (SouthernBiotech, Birmingham, AL, USA) or anti-His-RPE (Miltenyi Biotec, Bergisch Gladbach, Germany). For each marker, a “fluorescence minus one” (FMO) control was included. Acquisition of the samples was performed on a FACS Canto II (BD Biosciences), using the DIVA software and the Flowjo software (BD Biosciences) for analysis.

For sorting we employed freshly isolated PBMCs or defrosted lymph node and tonsil cells as previously described (3). First, T cells were depleted using the MACS system (Miltenyi Biotec) on an LD column after incubation with anti-CD3 (hybridoma clone PPT3/FyH2, kindly given by Dr. K. Haverson, University of Bristol, UK) and anti-mouse IgG magnetic beads (Miltenyi Biotec). The CD3-depleted fraction was then stained with anti-CD172a, anti-CADM1, anti-CD14 and anti-CD4 as described above, with the exception of V500-conjugated streptavidin which was replaced by streptavidin coupled to Alexa Fluor 750-APC (Thermofisher). Sorting of cDC1, cDC2, pDC and monocytes (for PBMCs) or CD14^high^ cells (for lymph nodes and tonsils) was performed using a FACS Aria (BD Bioscience) and the DIVA software.

### *In vitro* Stimulation of Sorted Blood Mononuclear Phagocytes With TLR Ligands

Following sorting, blood cDC1, cDC2, pDC and monocytes were plated in 96-well plates (Corning) at 5 × 10^4^ cells/well in 100 μL of DMEM supplemented with 10% FBS, 1% non-essential amino acids, 1% sodium pyruvate, 1% HEPES and 20 μM 2-mercapthoethanol (all Thermofisher, Gibco). cDC1, cDC2 and monocytes, were stimulated with 10 μg/ml PAM3Cys-SKKKK (PAM3Cys L2000, EMC microcollections, Tübingen Germany) or left unstimulated as a control, while pDC were stimulated with 10 μg/ml PAM3Cys, 10 μg/ml polyinosinic-polycytidytic acid (poly I:C, Sigma-Aldrich), 5 μg/ml gardiquimod (InvivoGen, San Diego, CA, USA), 5 μg/mL resiquimod (Sigma-Aldrich), or 5 μg/ml CpG oligodeoxynucleotide (CpG, sequence D32, ggTGCGTCGACGCAGggggg, Eurofins Genomics, Konstanz, Germany) or left unstimulated (controls). After 3 h of stimulation, cells were resuspended in 900 μL TRIzol (Thermofisher) and kept at −70°C until mRNA extraction.

### Collection and Processing of Lymph Nodes and Tonsils From Pigs Experimentally Infected With Classical Swine Fever Virus

The studies in pigs were performed in compliance with the Animal Welfare Act (TSchG SR 455), the Animal Welfare Ordinance (TSchV SR 455.1), and the Animal Experimentation Ordinance (TVV SR 455.163) of Switzerland, under license BE105/15. All experiments were reviewed by the cantonal committee on animal experiments of the canton of Bern and approved by the cantonal veterinary authority (Amt für Landwirtschaft und Natur LANAT, Veterinärdienst VeD, Bern, Switzerland). Fifteen 10-week-old Large White pigs, raised at the institute under SPF conditions, were used for this experiment. The fifteen animals, including both males and females, were assigned randomly to three experimental groups: two groups of six animals to be infected with CSFV, and one mock control group of three animals. Two CSFV genotype 1 strains differing in virulence were used for infection: the highly virulent CSFV strain vEy-37 (7), and the low virulent Pinar del Rio strain (8). The CSFV strain vEy-37 was prepared from PEDSV.15 cells transfected with transcripts from the cDNA clone pEy-37 as described elsewhere (16). The CSFV strain Pinar del Rio was propagated on PEDSV.15 cells using virus-positive serum collected 4 weeks post-infection from a persistently infected piglet (piglet #7) (17). In order to remove any soluble factors from the virus stocks, the PEDSV.15 CSFV extracts were purified by ultracentrifugation at 70,000 × g for 4 h through a 10% sucrose cushion and the pellets were resuspended in MEM. The six pigs of the two infection groups were then inoculated intranasally with 10^6^ TCID_50_/pig of purified vEy-37 or Pinar del Rio in 5 mL MEM. The 3 pigs of the control group were administered 5 mL MEM intranasally. At 18 h post-infection, three animals from each infected group were euthanized and tonsils were harvested. At 42 h post-infection, the remaining three pigs of each group were euthanized, and mandibular and retropharyngeal lymph nodes were harvested. Tonsils and mandibular and retropharyngeal lymph nodes from the three control animals were harvested the day after. Lymph nodes and tonsils were processed as described above. Cells were then frozen in liquid nitrogen at 10^7^ cells/mL in freezing medium until further use. After thawing, cells were sorted by flow cytometry as described above and harvested in TRIzol to perform mRNA sequencing on the cDC1, cDC2, pDC, and CD14^high^ subsets of both control and infected animals. TRIzol samples were kept at −70°C until further use.

### RNA-Seq and Bioinformatic Processing

For RNA-Seq, mRNA was extracted from TRIzol lysates of FACS-sorted cDC1, cDC2, pDC and monocyte or CD14^high^ subsets from PBMC, lymph nodes and tonsils using the Nucleospin RNA kit (Macherey Nagel, Düren, Germany) with a modified protocol as described previously (3). Briefly, 0.2 mL chloroform was added to 1 mL of thawed TRIzol lysate, and after vigorous shaking and following a 12,000 × g centrifugation at 4°C for 15 min, the aqueous phase was harvested. 0.5 mL 75% ethanol was added and the RNA precipitated for 10 min at room temperature before loading on a NucleoSpin RNA column. RNA extraction was then carried on following the manufacturer's instructions, including a DNase step. RNA was eluted from the column with 40 μL RNase-free water and the quality and quantity of the extracted RNA was determined using an Agilent 2100 Bioanalyzer (Agilent Technologies, Santa Clara, CA, USA) and a Qubit 2.0 Fluorometer (Thermofisher). All the RNA samples used for this study were of high quality (RNA integrity number RIN > 8) and libraries were prepared with TruSeq RNA Library Prep Kit v2 (Illumina). Total mRNA libraries were randomly multiplexed with 8 samples per lane and sequenced on the Illumina HiSeq2500 platform. The Illumina BCL output files with base calls and qualities were converted into FASTQ file format and demultiplexed with the CASAVA (v1.8.2) software. The RNAseq data are available in the European Nucleotide Archive (http://www.ebi.ac.uk/ena) under the accession numbers PRJEB37564 and PRJEB37565 for the *in vitro* and *in vivo* activated DC, respectively.

For the blood mononuclear cell subsets incubated with or without TLR ligands, between 19.2 and 33.3 million single-end reads were available per sample. For the tonsil and lymph node subsets, between 28.1 and 37.0 million single-end reads were available per sample. The quality of the data was assessed using fastqc v.0.11.5 and RSeQC v. 2.6.4 (18). The reads were mapped to the pig reference genome (assembly Sscrofa11.1) using HiSat2 v. 2.1.0 (19). FeatureCounts v. 1.6.0 (20) was used to count the number of reads overlapping with each gene as specified in the Ensembl annotation build 91. Differential gene expression analysis was performed with DESeq2 v. 1.18.1 (21) in R v. 3.4.2. For each of the TLR-stimulated blood mononuclear cell subsets, we compared stimulated cells to their corresponding unstimulated control. For the PAM3Cys-stimulated samples, we also performed pairwise comparisons of cell subsets. For pDC, the samples from the different TLR stimulations were also compared between each other.

To compare gene expression profiles of DC subtypes across tissues, the data from lymph node and tonsil populations were combined with previously generated RNA-Seq data from blood (3). For consistency, older datasets were reanalyzed as described above. The cell-type specific transcription signatures of the different subsets were compared as described previously (22). For each tissue, subtype-specific gene expression signatures were obtained by performing pairwise tests of differential gene expression between each cell type and a pool of all remaining cell types. All gene lists were then sorted based on FDR-adjusted *p*-values to have the most highly upregulated genes at the top and the most strongly downregulated genes at the bottom.

Each cell type-specific signature from a given tissue was compared with all signatures from a second tissue using the R package OrderedList v. 1.44.0 (23). This tool determines the number of shared elements in the tails of two lists and calculates a final similarity score where genes receive more weight the closer they are to the top or bottom of the list. This ensures that the score is dominated by the genes showing the most significant differential expression. We report similarity scores based on *n* = 1,000 genes each from the top and bottom of the lists. The relative similarity among the cell types was generally consistent for other values of n (assessed for values between 100 and 2500). To assess the statistical significance of the similarity scores, the observed values were compared with a null distribution obtained by reshuffling the genes. Because invariant genes do not influence the similarity score, the middle 60% of genes were excluded from the permutations.

The molecular signatures of *in vivo* activated DC were also analyzed by “ranked gene set enrichment analysis” (GSEA) (24) using a selection of blood transcription modules (BTM) defined by Li et al. (25) and modified for pigs as previously described (26). For this selection, all cell-type specific and non-classified BTM [“TBA” in Li et al. (25)] were omitted resulting in 86 BTM (M4.0, M4.1, M4.2, M4.3, M4.4, M4.5, M4.6, M4.7, M4.8, M4.10, M4.12, M5.0, M6, M13, M14, M15, M16, M22.0, M22.1, M23, M24, M25, M27.0, M27.1, M28, M29, M33, M34, M37.3, M38, M39, M40, M43.0, M43.1, M50, M51, M53, M59, M64, M65, M67, M68, M71, M75, M76, M77, M78, M86.0, M86.1, M92, M95.0, M95.1, M103, M109, M111.0, M111.1, M112.0, M112.1, M114.1, M115, M119, M122, M127, M129, M138, M139, M143, M144, M146, M147, M150, M158.0, M158.1, M165, M168, M169, M200, M209, M216, M219, M225, M226, M230, M250, S10, S11), which mainly inform on antigen presentation, inflammation, interferon (IFN) type I responses, metabolic processes and cell cycle. To compare the module activity between highly and low virulent CSFV infection, normalized enrichment scores (NES) of significantly enriched modules (false discovery rate, *q*-value < 0.05) were displayed.

## Results

### Transcription Profiles of Blood cDC1, cDC2, pDC and Monocytes Following PAM3Cys Stimulation

**Figure 1:**
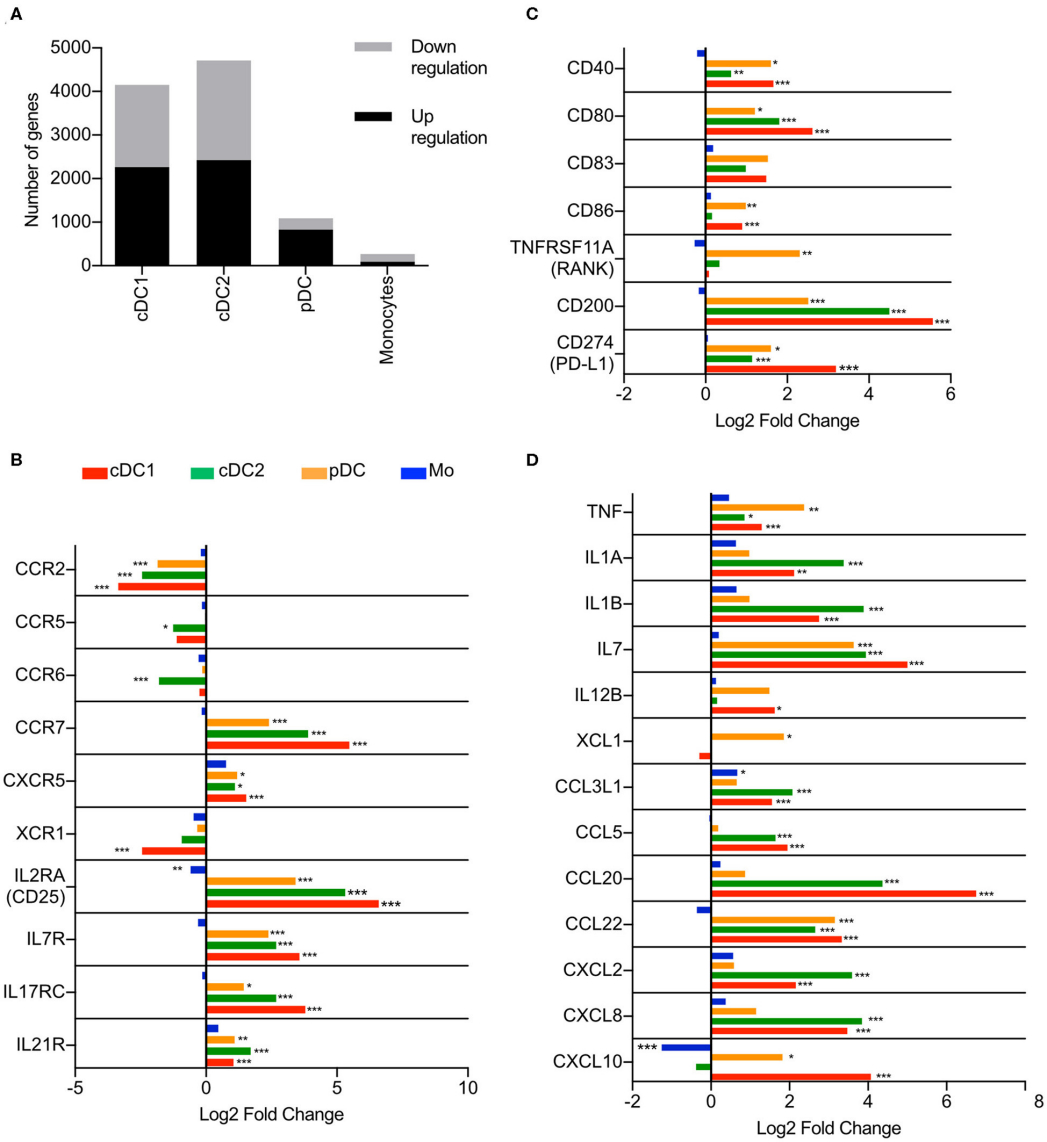
Transcription profiles of porcine blood mononuclear phagocytes following PAM3Cys stimulation. cDC1, cDC2, pDC and monocytes from three different animals were sorted by FACS and stimulated for 3 h with 10 μg/ml of PAM3Cys or left unstimulated as control. **(A)** Number of significantly regulated genes (padj < 0.05) when comparing PAM3Cys-stimulated cells to the corresponding non-stimulated control. **(B)** Expression of selected chemokine and cytokine receptor genes as log2 fold change between PAM3Cys-stimulated cells and their corresponding non-stimulated control. **(C)** Expression of selected costimulatory molecule genes as log2 fold change between PAM3Cys-stimulated cells and their corresponding non-stimulated control. **(D)** Expression of selected chemokine and cytokine genes as log2 fold change between PAM3Cys-stimulated cells and their corresponding non-stimulated control. DESeq2 analysis * padj < 0.05, ** padj < 0.01, *** padj < 0.0001.

As a first step, we applied RNA-Seq to characterize, in a comparative manner, the activation profiles of sorted blood DC including cDC1, cDC2, and pDC as well as monocytes, all defined as previously described (3). Due to limitations in cell numbers of the rare cDC1 subset following sorting, we focused on the stimulation of the different populations with the TLR1/TLR2 agonist PAM3Cys which was selected based on its ability to directly activate all four targeted subsets (3). When assessing the number of significantly (padj < 0.05) up- and downregulated genes, cDC1 and cDC2 subsets appeared to be the most responsive to PAM3Cys stimulation, with over 4000 differentially regulated genes (Figure 1A). Over 1000 genes were differentially regulated in pDC following PAM3Cys stimulation, comprising mostly upregulated genes (827 upregulated and 265 downregulated). In monocytes, despite a higher expression of *TLR1* and *TLR2* at homeostasis (3), a lower amount of differentially regulated genes (93 upregulated and 176 downregulated) was observed (Figure 1A).

A switch in chemokine receptor expression is a hallmark of DC activation (2). Following PAM3Cys stimulation of DC but not monocytes, we observed a significant decrease of *CCR2* mRNA, combined with a significant increase of *CCR7* mRNA, the latter coding for the key chemokine receptor responsible for migration to the lymph nodes (Figure 1B). All three DC subsets displayed also an increase in *CXCR5* expression (Figure 1B), a receptor involved in the migration toward B-cell follicles in the lymph nodes (2). Only cDC2 displayed a significant decrease in *CCR5* and *CCR6* expression. Interestingly, stimulation of DC but not monocytes also induced upregulation of a number of cytokine receptor genes, including *IL2RA, IL7R, IL17RC*, and *IL21R* (Figure 1B), some of which encode receptors for T-cell cytokines.

Co-stimulatory molecules *CD40, CD80*, and *CD83* were upregulated in all three DC subsets but not in monocytes after stimulation, while only cDC1 and pDC significantly increased their *CD86* expression (Figure 1C). The inhibitory molecules *CD200* (OX-2) and *CD274* (PD-L1) were also upregulated in all three DC subsets.

The PAM3Cys stimulation also induced the expression of several cytokine and chemokine genes. The three DC subsets significantly upregulated *TNF, IL7* and *CCL22* (Figure 1D), the latter encoding a chemokine attracting Th2 and regulatory T cells (27). The two cDC displayed a significant increase in *IL1A, IL1B*, and in a broad range of chemokine genes (*CCL3L1, CCL5, CCL20, CXCL2*, and *CXCL8*). Conventional DC type 1 and pDC were the only subsets showing an increase in *CXCL10*, while only cDC1 significantly upregulated *IL12B* (Figure 1D). With respect to cytokines, monocytes were not very responsive to PAM3Cys stimulation, with a significant upregulation of only *CCL3L1*.

Stimulation with PAM3Cys also had an impact on PRR expression by porcine DC and monocytes (Supplementary Figure 1). The receptors for PAM3Cys *TLR1* and *TLR2* were upregulated by cDC1 and cDC2, and by pDC and cDC2, respectively. Both cDC subsets also significantly upregulated their expression of *TLR10*, while their expression of *TLR3, TLR4, TLR8*, and *TMEM173* (STING) was downregulated. Stimulation with PAM3Cys also induced a significant upregulation of the RIG-I like receptors (RLR) *DDX58* (RIG-I) and *IFIH1* (MDA5), as well as of the NOD-like receptor (NLR) *NLRP3* by pDC. Although no changes were observed for TLR expression in monocytes, their expression of *DHX58* (LGP2), *DDX58* and *IFIH1* was downregulated (Supplementary Figure 1A). In most cases, the TLR stimulation promoted the expression of many signaling molecules of the NFκB and JAK-STAT pathways as well as of PRR adaptor molecules (Supplementary Figures 1B,C). Interestingly, these responses were seen in DC but not in monocytes.

### Transcriptional Profile of pDC After Stimulation With Different TLR Ligands

**Figure 2:**
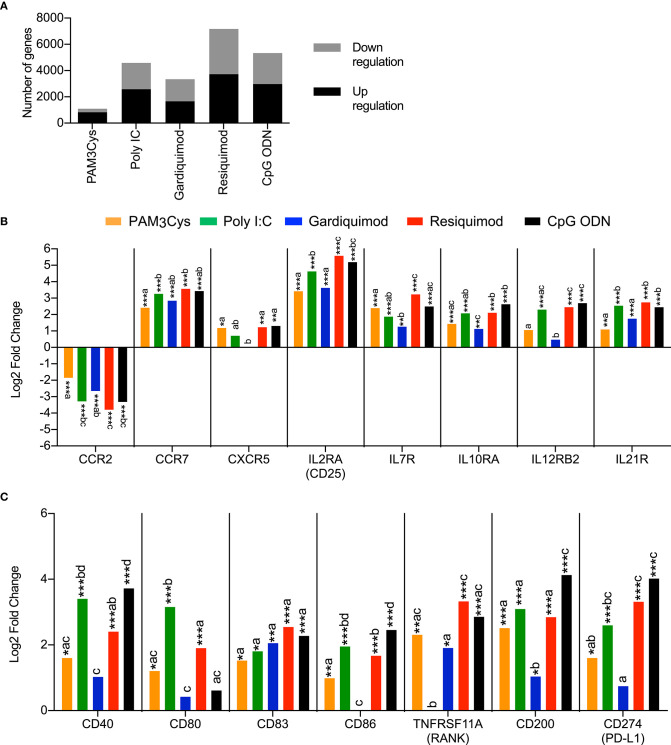
Transcription profiles of porcine blood pDC stimulated with TLR ligands. pDC from three different animals were sorted by FACS and stimulated with 10 μg/ml of PAM3Cys, 10 μg/ml poly I:C, 5 μg/ml gardiquimod, 5 μg/ml resiquimod, 5 μg/ml CpG ODN D32, or were left unstimulated as control. **(A)** Number of significantly regulated genes (Padj < 0.05) when comparing TLR ligand-stimulated pDC to control pDC. **(B)** Expression of selected chemokines and cytokines receptor genes as log2 fold change between TLR ligand-stimulated pDC and control pDC. **(C)** Expression of selected costimulatory molecule genes as log2 fold change between TLR ligand-stimulated pDC and control pDC. DESeq2 analysis * padj < 0.05, ** padj < 0.001, *** padj < 0.0001. For each gene, different letters indicate statistical significance between two TLR-ligand stimulation groups as calculated by DESeq2 analysis.

In a previous study, we showed that blood pDC were activated by a wide range of TLR ligands and were key cells in the activation of cDC after TLR7 and TLR9 stimulation (3). Therefore, we were particularly interested in comparing immune response profiles of porcine pDC to different TLR ligands. In parallel to the PAM3Cys stimulation shown in Figure 1, sorted pDC from the same animals were also stimulated with the TLR ligands poly I:C (TLR3), gardiquimod (TLR7), resiquimod (TLR7/8), or CpG ODN (TLR9), recognized by receptors highly expressed in porcine pDC. These four ligands were all more efficient than PAM3Cys in inducing either up- or downregulation of genes following stimulation, when compared to the unstimulated control (Figure 2A). Resiquimod induced the strongest changes in gene expression, followed by CpG ODN and poly I:C.

In terms of activation markers, all TLR ligands induced a significant downregulation of *CCR2* mRNA and a significant upregulation of *CCR7* mRNA (Figure 2B). Transcription of *CXCR5* was also induced by all TLR ligands with the exception of gardiquimod. Stimulation with TLR ligands also had a potent impact on cytokine receptor expression, inducing transcription of *IL2RA, IL7R, IL10RA, IL12RB2*, and *IL21R*. In many cases the strongest effects were found with poly I:C, resiquimod and CpG ODN (Figure 2B).

Stimulation with PAM3Cys, poly I:C, resiquimod and CpG ODN also induced the upregulation of several costimulatory molecules such as *CD40, CD80, CD83, CD86*, and *TNFRSF11A* (RANK), with the noticeable exception of *CD80* following CpG ODN stimulation and *TNFRSF11A* following poly I:C stimulation (Figure 2C). These four TLR ligands also induced a significant transcriptional upregulation of *CD200* and C*D274*, both coding for T-cell inhibitory molecules.

**Figure 3:**
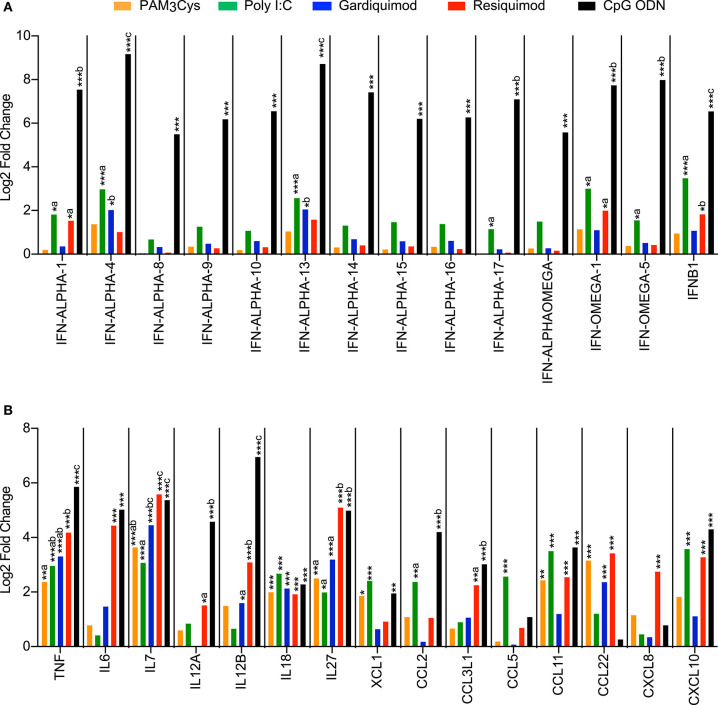
Transcription profiles of porcine blood pDC stimulated with TLR ligands. pDC from 3 different animals were sorted by FACS and stimulated with 10 μg/ml of PAM3Cys, 10 μg/ml poly I:C, 5 μg/ml gardiquimod, 5 μg/ml resiquimod, 5 μg/ml CpG ODN D32, or were left unstimulated as control. **(A)** Expression of selected type I interferon genes as log2 fold change between TLR ligand-stimulated pDC and control pDC. **(B)** Expression of selected cytokine and chemokine genes as log2 fold change between TLR ligand-stimulated pDC and control pDC. DESeq2 analysis * padj < 0.05, ** padj < 0.001, *** padj < 0.0001. For each gene, different letters represent statistical significance between two TLR-ligand stimulation groups as calculated by DESeq2 analysis.

A broad range of cytokine and chemokine genes was upregulated in pDC following TLR-ligand stimulation (Figures 3A,B). CpG ODN was found to be the main inducer of many type I interferon genes (10 IFNα genes, 3 IFNω genes, and IFNβ1) (Figure 3A). Poly I:C and resiquimod also increased the expression of *IFN-ALPHA-1, IFN-ALPHA-13, IFN-OMEGA-1*, and *IFNB1* genes, but to a lesser extent than CpG ODN. Stimulations with Poly I:C, resiquimod and CpG ODN all induced a significant upregulation of *CXCL10*, a chemokine involved in the recruitment of activated T- and B lymphocytes and a well-known IFN-responsive gene (28). Several other chemokines were also upregulated following stimulation, with unique expression profiles for different TLR ligands (Figure 3B). CpG ODN and resiquimod were the only ligands inducing a significant upregulation of the genes coding for IL-12p35 and IL-12p40 (*IL12A* and *IL12B*), thus suggesting the production of the Th1-promoting cytokine IL-12p70 by pDC. Transcription of the pro-Th1 cytokine gene *IL18* was increased following treatment by all ligands. The expression of *IL7*, known to be essential for lymphocyte development and homeostasis (29), and *IL27*, implied in regulating T-cell responses (30), was induced by all TLR ligands tested, with resiquimod and CpG ODN inducing higher levels (Figure 3B).

The expression of PRR was differentially regulated, depending both on the type of PRR and on the type of stimulus. A significant downregulation of *TLR1, TLR6, TLR9*, and *TLR10* was seen irrespective of the TLR ligand used for stimulation (Supplementary Figure 2A). For *TLR3* and *TLR7* expression, a significant upregulation was observed following stimulation with poly I:C and CpG ODN, while resiquimod induced a downregulation of these genes. A similar pattern was observed for *TLR8* expression, but only the downregulation induced by resiquimod stimulation was found to be significant. The RLRs *DHX58, DDX58*, and *IFIH1* were all upregulated after PAM3Cys and CpG ODN treatment, and poly I:C induced an even more significant upregulation of *DHX58* and *DDX58* expression. Transcription of *NLRP3* was enhanced by all TLR ligands, with poly I:C, resiquimod and CpG ODN inducing again the strongest effect (Supplementary Figure 2A). We also found a profound stimulatory effect of all TLR ligands on certain signaling molecules of the NFκB pathway and the JAK-STAT pathway as well as on some PRR adaptor molecules (Supplementary Figures 2B,C).

### Identification of cDC1, cDC2, and pDC in Lymphoid Tissue by Flow Cytometry

Having characterized the immune response profiles of DC subsets after *in vitro* stimulation, we wanted to validate this data using an *in vivo* CSFV infection model. In particular, we were interested whether the observations made *in vitro* with TLR-stimulated blood DC were comparable to those found in lymphoid tissue DC subsets from infected animals.

**Figure 4:**
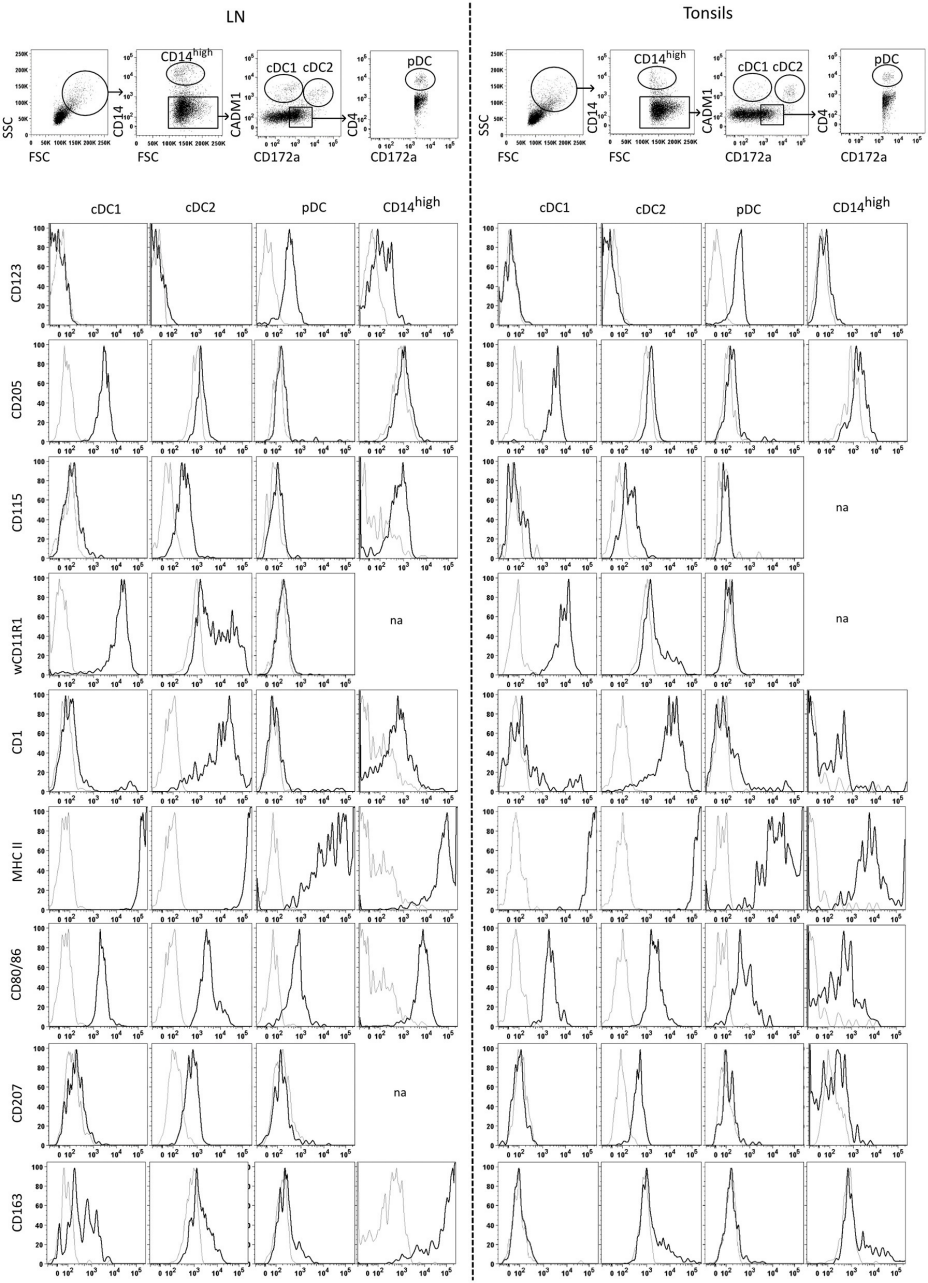
Identification and phenotyping of tonsil and mandibular lymph node mononuclear phagocytes isolated from tonsils and lymph nodes. The top panel shows the gating strategy following multicolor FCM staining using antibodies against CD14, CD172a, CADM1, and CD4. After excluding doublets, cells high in FSC/SSC were gated, followed by gating on the CD14^high^ population. Among the CD14^−^ cells, cDC1 were gated as CD14^−^ CD172a^−/low^ CADM1^+^, cDC2 as CD14^−^CD172a^+^CADM1^+^, and pDC as CD14^−^CD172a^+^CADM1^+^CD4^+^. The cell-surface expression of CD123, CD205, CD115, wCD11R1, CD1, MHC II, CD80/86, CD207 and CD163 was assessed for cDC1, cDC2, pDC and monocytic cells. Histograms show staining for each of the sub-populations with the corresponding FMO control (gray histograms). Across tissues, the profiles displayed for a given marker were obtained from the same animal and are representative of three independent experiments using three different animals. “na” no data available due to low number of cells.

Prior to infection experiments, in-depth phenotypic analyses were performed in order to ensure proper identification of porcine DC subsets in lymphoid tissues by flow cytometry. To this end, we harvested tonsils and mandibular lymph nodes from SPF pigs and performed the same staining protocol employed to define DC subsets in porcine blood (3). After gating on cells with a higher FSC and SSC, we defined monocytic cells as CD14^high^, and gated on two CD172a/CADM1-defined subsets within the CD14 negative fraction, with the putative cDC1 being CD14^−^CD172a^−^CADM1^+^, and the putative cDC2 being CD14^−^CD172a^+^CADM1^+^. For pDC, we gated on CD14^−^CD172^+^CADM1^−^ cells, followed by a gate on CD4^+^ cells (Figure 4, top panels). Using this gating, we investigated the expression of a range of surface markers on each of these subsets. Most of these markers had been used previously to characterize porcine DC subsets from blood and we observed similar expression patterns for DC subsets from tonsils and mandibular lymph nodes. As in the peripheral blood, putative pDC in lymph nodes and tonsils were the only subset to express CD123 (IL3RA). Putative cDC1 expressed high levels of CD205 and wCD11R1 while putative cDC2 expressed CD1, CSF1R (CD115) and CD207. All DC populations expressed class II MHC and costimulatory molecules CD80/86, with both putative cDC subsets expressing very high levels of MHC II, and higher levels of CD80/86 when compared to pDC. The CD14^high^ monocytic cell subset was phenotypically similar to cDC2, but expressed lower levels of CD1 and higher levels of CD115. In the mandibular LN, but not the tonsils, monocytic cells also expressed high levels of CD163 (Figure 4).

### Transcriptional Characterization of DC Subsets Isolated From Secondary Lymphoid Organs

**Figure 5:**
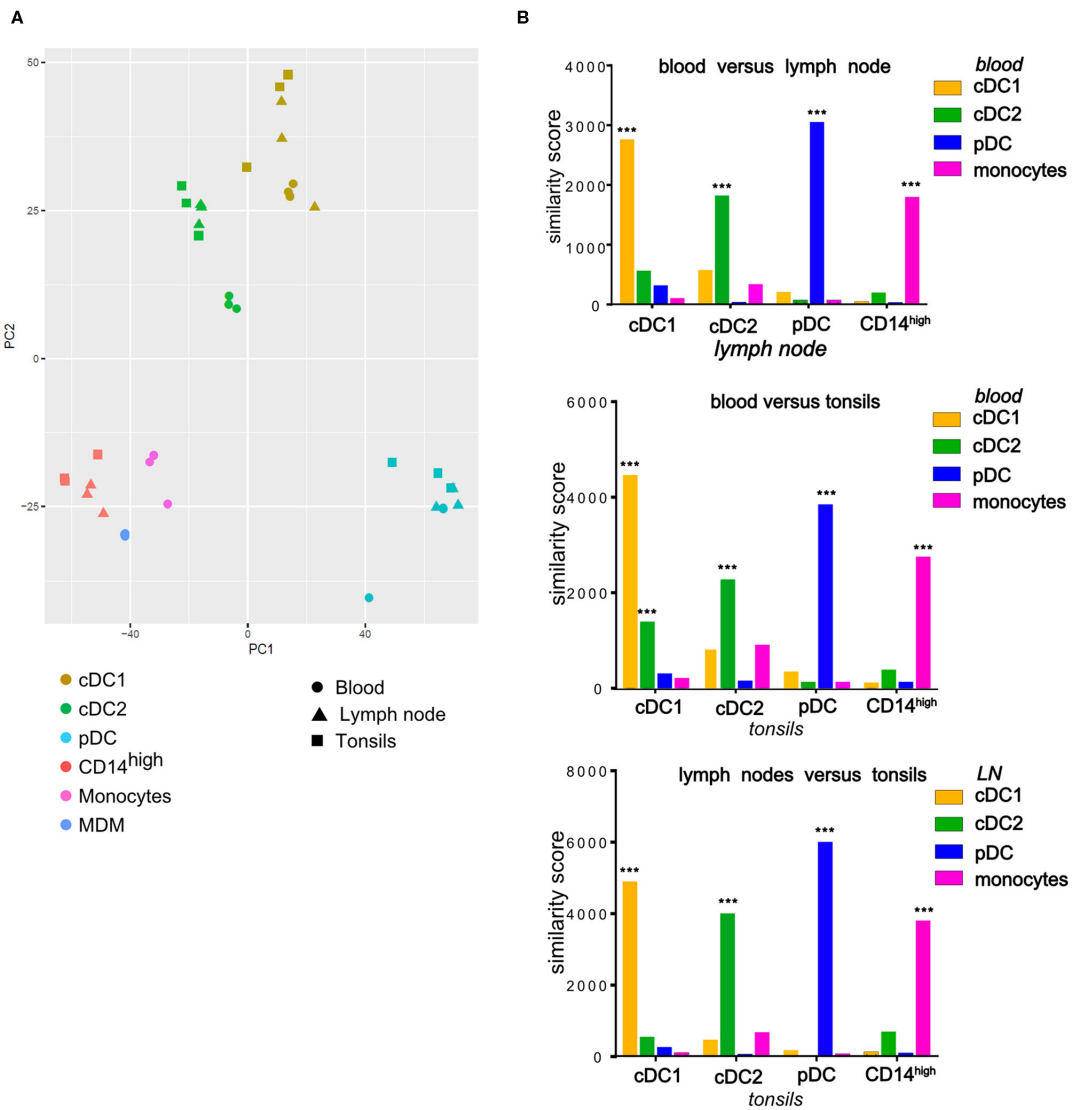
Comparison of transcription profiles of mononuclear phagocytes isolated from tonsils and LN (cDC1, cDC2, pDC, CD14^high^) with their blood counterparts (cDC1, cDC2, pDC, monocytes) as well as with monocyte-derived macrophages (MDM) at homeostasis. Cells isolated from porcine tonsils or lymph nodes were sorted by FACS and their RNA was sequenced. **(A)** Plot of the first 2 axes from a principal component analysis (PCA) based on the 500 most variable genes. For each compartment (blood, tonsils, lymph nodes), data were obtained from three different animals. **(B)** Each cell-type specific signature was compared to all signatures of subsets from a second tissue and a similarity score was calculated. To assess the statistical significance of the similarity scores, the observed values were compared to a null distribution obtained by reshuffling the genes. *** = empirical *p*-value < 0.001.

To verify the proposed identity of the phenotypically defined subsets, we FACS-sorted these cells using the gates described in Figure 4 and performed RNA-Seq. For the LN, we pooled cells from mandibular and retropharyngeal LN to obtain sufficient amounts of RNA. The transcriptomic data confirmed that only the cDC1, cDC2, and pDC subsets but not the CD14^high^ subset expressed the *bona fide* DC-specific marker *FLT3*, and the expression of *CD14, CADM1*, and *CD4* matched with what was observed by flow cytometry (Supplementary Figures 3A,B for LN and tonsils, respectively). Making use of the data we published previously with blood DC subsets and monocytes (3), we performed a principal component analysis on all samples using the 500 most variable genes (Figure 5A). Regardless of the compartment the cells were isolated from (blood, tonsils or LN), cDC1, cDC2 and pDC were clustering with their counterparts from the other tissues, and CD14^high^ cells clustered with monocytes and monocyte-derived macrophages (MDM). This was confirmed by computing the organ-dependent similarity scores for the transcriptional profiles of each cell-type, which included the following comparisons: blood vs. LN, blood vs. tonsils and LN vs. tonsils (Figure 5B). For both, tonsils and LN cells, the transcriptional signature of each DC subset was similar to the corresponding blood DC subset. The only exception was that cDC1 from tonsils also shared some similarity with blood cDC2. In addition, the CD14^high^ population showed high transcriptional similarity to monocytes, but no significant similarity to any of the DC subsets.

Finally, to confirm the correct identification of DC subsets, we investigated a number of genes known to be differentially expressed in a subset-dependent manner. Table 1 shows selected genes that were expressed specifically either by all three DC subsets (cDC1, cDC2, and pDC) or by both cDC subsets in tonsils and LN. All three DC subsets specifically expressed the pan-DC markers *FLT3* and *BCL11A*, and the gene coding for vinculin (*VCL*), an important component of podosomes involved in antigen sampling (31). The expression pattern of *IRF4* and *IRF8* matched what was described in mouse or human, with pDC co-expressing both genes and cDC1 and cDC2 expressing mainly *IRF8* and *IRF4*, respectively. Four genes found to be specifically expressed by both cDC subsets in porcine blood, namely *NAV1, PAK1, SIGIRR*, and *NAPSA*, were also overexpressed by cDC1 and cDC2 subsets from secondary lymphoid organs. The highly dominant expression of cross-species cDC1-specific genes including *BATF3, XCR1, ANPEP* (coding for CD13), *DPP4* (CD26), *GCSAM, CLNK, SNX22*, or *RAB7B* (Table 2) confirmed the correct identification of this subset in porcine lymphoid tissue. In addition, genes highly over-expressed in cDC1 in porcine blood such as *SCIMP, CD34*, or *CD226* were also found in lymphoid tissue cDC1. Interestingly, we also found high levels of *ITGAE* (CD103) transcripts in this subset, contrasting with porcine peripheral blood cDC1 (3). The integrin subunit CD103 is well-described in other species as a marker for cDC1 at mucosal surfaces and in secondary lymphoid organs (32).

**Table 1:** cDC-specific and DC-specific differentially regulated genes.

**Gene**		**cDC1**	**cDC2**	**pDC**	**CD14^**high**^**
*FLT3*	LN	9,506 ± 4,257^a^	8,096 ± 674	7,997 ± 404	128 ± 35
	Tonsils	16,282 ± 4,106	7,216 ± 2,406	7,224 ± 944	152 ± 104
*BCL11A*	LN	1,631 ± 153	1,587 ± 258	8,943 ± 108	360 ± 102
	Tonsils	2,295 ± 189	2,580 ± 351	8,313 ± 560	570 ± 386
*VCL*	LN	810 ± 23	3,137 ± 59	4,028 ± 205	278 ± 78
	Tonsils	1,460 ± 142	2,940 ± 952	3,115 ± 675	535 ± 43
*IRF4*	LN	1,432 ± 730	1,631 ± 192	6,126 ± 687	536 ± 180
	Tonsils	1,407 ± 624	2,078 ± 448	6,195 ± 1,332	876 ± 563
*IRF8*	LN	58,913 ± 23,400	3,323 ± 595	1,09,432 ± 2,441	7,592 ± 1,161
	Tonsils	69,736 ± 11,350	8,346 ± 926	1,08,212 ± 13,113	11,944 ± 1,550
*SIGIRR*	LN	283 ± 172	543 ± 148	7 ± 4	84 ± 14
	Tonsils	244 ± 100	585 ± 27	96 ± 77	163 ± 120
*PAK1*	LN	8,001 ± 2,785	33,717 ± 1,396	20 ± 3	4,484 ± 1,040
	Tonsils	14,883 ± 3,505	30,987 ± 7,543	120 ± 155	6,162 ± 1,166
*NAV1*	LN	1,533 ± 571	2,005 ± 60	388 ± 60	1,107 ± 395
	Tonsils	1,983 ± 340	1,903 ± 675	298 ± 127	683 ± 44
*NAPSA*	LN	1,324 ± 398	2,164 ± 611	70 ± 50	79 ± 51
	Tonsils	2,069 ± 117	2,926 ± 249	348 ± 320	418 ± 311

a *Mean number of reads ± SD*.

**Table 2:** Differentially regulated genes in cDC1.

**Gene**		**cDC1**	**cDC2**	**pDC**	**CD14^**high**^**
*XCR1*	LN	12,854 ± 5,720^a^	22 ± 18	147 ± 149	106 ± 23
	Tonsils	16,581 ± 1,297	52 ± 51	769 ± 634	743 ± 494
*BATF3*	LN	2,016 ± 728	787 ± 99	2 ± 2	462 ± 166
	Tonsils	2,920 ± 990	512 ± 155	12 ± 8	1,510 ± 589
*ANPEP*	LN	28,706 ± 12,442	484 ± 134	10 ± 3	47 ± 43
(CD13)	Tonsils	2,920 ± 990	512 ± 155	12 ± 8	1,510 ± 589
*DPP4*	LN	2212 ± 1003	933 ± 160	1,225 ± 667	88 ± 10
(CD26)	Tonsils	34755 ± 8044	1164 ± 444	9 ± 13	233 ± 144
*SCIMP*	LN	5,261 ± 455	133 ± 20	102 ± 75	469 ± 309
	Tonsils	8,820 ± 566	2,528 ± 894	1,005 ± 591	1,233 ± 1,018
*ITGAE*	LN	2,927 ± 1,135	103 ± 55	144 ± 42	92 ± 27
(CD103)	Tonsils	4,482 ± 790	1,046 ± 366	245 ± 18	210 ± 118
*CD226*	LN	1,408 ± 504	45 ± 28	3 ± 3	6 ± 6
	Tonsils	903 ± 225	15 ± 15	6 ± 9	4 ± 5
*CD34*	LN	1,809 ± 714	310 ± 9	284 ± 59	194 ± 153
	Tonsils	2,890 ± 493	414 ± 181	206 ± 99	50 ± 18
*GCSAM*	LN	1,154 ± 246	51 ± 18	19 ± 16	25 ± 20
	Tonsils	1,962 ± 369	226 ± 198	278 ± 183	335 ± 175
*RAB7B*	LN	1,093 ± 356	5 ± 5	6 ± 4	60 ± 21
	Tonsils	1,080 ± 155	5 ± 3	3 ± 3	191 ± 75
*CLNK*	LN	3,052 ± 1,837	7 ± 0	0	4 ± 6
	Tonsils	2,695 ± 556	3 ± 5	0	5 ± 5
*SNX22*	LN	1,007 ± 312	39 ± 18	22 ± 9	13 ± 2
	Tonsils	1520 ± 351	53 ± 9	31 ± 11	22 ± 19

a * Mean number of reads ± SD*.

Table 3 displays selected genes that were overexpressed specifically by the cDC2 subset and confirm their identity. As for their counterparts in porcine blood, *FCER1A* and *NOTCH4* were specifically expressed by the cDC2 subset in both lymph nodes and tonsils. Interestingly, transcription of *MS4A2*, coding for the β subunit of the high-affinity IgE receptor, as well as transcription of *CD207* (Langerin) were found to be restricted to the cDC2 subset. The cDC2-associated genes *CD1E* (33) and *SLCO3A1* (34) and genes associated with migratory DC or lymphoid-organ DC such as *FSCN1* (35) and *PLA2G2D* (36) were also expressed in porcine cDC2 from secondary lymphoid tissues.

**Table 3:** Differentially regulated genes in cDC2.

**Gene**		**cDC1**	**cDC2**	**pDC**	**CD14^**high**^**
*FCER1A*	LN	5 ± 0^a^	241 ± 114	54 ± 59	15 ± 18
	Tonsils	20 ± 16	295 ± 112	67 ± 67	13 ± 16
*MS4A2*	LN	638 ± 152	32,459 ± 3,533	979 ± 995	5,660 ± 4,419
(*FCER1B*)	Tonsils	1,602 ± 860	24,433 ± 8,617	304 ± 200	1,590 ± 130
*NOTCH4*	LN	1,590 ± 344	5,688 ± 1,676	239 ± 45	777 ± 333
	Tonsils	2,104 ± 582	6,706 ± 1,972	258 ± 42	838 ± 479
*CD207*	LN	6 ± 3	3,450 ± 569	1 ± 0	89 ± 21
	Tonsils	17 ± 23	1,519 ± 827	0 ± 0	42 ± 23
*FSCN1*	LN	1,130 ± 45	6,936 ± 3,973	48 ± 49	3,290 ± 1,425
	Tonsils	846 ± 279	3,799 ± 985	99 ± 51	1,424 ± 228
*CD1E*	LN	120 ± 45	6,178 ± 2,671	4 ± 1	2,539 ± 912
	Tonsils	130 ± 112	10,845 ± 3,570	53 ± 63	812 ± 562
*SLCO3A1*	LN	1,043 ± 264	3,467 ± 280	972 ± 88	584 ± 262
	Tonsils	815 ± 207	4,003 ± 634	946 ± 223	361 ± 167
*PLA2G2D*	LN	339 ± 4	3,374 ± 1,956	12 ± 15	789 ± 894
	Tonsils	1,211 ± 625	10,317 ± 4,997	57 ± 54	1,579 ± 1,088

a *Mean number of reads ± SD*.

Selected genes expressed specifically in pDC are shown in Table 4, again confirming their correct identification in lymphoid tissue. The expression of the two pDC-specific transcription factors *TCF4* (E2-2) and *RUNX2* was restricted to the pDC subset. As with their blood counterparts, we found a subset-specific expression of *CD36, PLAC8, BLNK, LRP8, NOTCH3, TRAF4, CLEC12A, C2, C3*, and *CD93* in pDC from lymphoid tissues. *CMKLR1*, encoding the receptor for chemerin, which is specifically expressed by pDC in human blood (37), was also expressed in pDC from LN and tonsils.

**Table 4:** Differentially regulated genes in pDC.

**Gene**		**cDC1**	**cDC2**	**pDC**	**CD14^**high**^**
*TCF4*	LN	1,523 ± 524^a^	886 ± 66	22,483 ± 1,668	1,439 ± 130
(E2-2)	Tonsils	1,843 ± 368	1,677 ± 412	19,428 ± 4,631	1,947 ± 291
*RUNX2*	LN	16 ± 1	379 ± 50	869 ± 37	87 ± 31
	Tonsils	41 ± 3	152 ± 105	590 ± 174	116 ± 23
*CD8B*	LN	2 ± 1	1 ± 1	3942 ± 657	10 ± 9
	Tonsils	15 ± 21	9 ± 13	8,370 ± 1,635	4 ± 5
*CD36*	LN	936 ± 450	163 ± 39	23,382 ± 505	113 ± 17
	Tonsils	1,857 ± 327	175 ± 48	21,284 ± 1,889	35 ± 22
*CLEC12A*	LN	248 ± 92	46 ± 12	2,886 ± 347	20 ± 9
	Tonsils	334 ± 42	133 ± 67	2,854 ± 1,087	82 ± 96
*UNC93B1*	LN	3,632 ± 1,096	6,277 ± 222	15,497 ± 1,169	5,734 ± 535
	Tonsils	4,668 ± 1,279	6,560 ± 1,604	16,275 ± 2,951	6,025 ± 2,452
*NOTCH3*	LN	2 ± 1	5 ± 3	2,137 ± 182	247 ± 231
	Tonsils	0	11 ± 9	2,106 ± 675	1 ± 1
*TRAF4*	LN	1,591 ± 577	652 ± 407	6,334 ± 382	157 ± 90
	Tonsils	1,387 ± 332	1,004 ± 315	6,485 ± 856	502 ± 381
*PLAC8*	LN	686 ± 211	999 ± 65	10,817 ± 187	1,885 ± 1,454
	Tonsils	819 ± 315	1,132 ± 327	11,223 ± 1,654	819 ± 274
*BLNK*	LN	1,551 ± 1,024	714 ± 48	23,751 ± 2,093	2,657 ± 259
	Tonsils	1,474 ± 427	1,616 ± 567	27,304 ± 3,353	1,810 ± 349
*LRP8*	LN	612 ± 275	929 ± 123	12,756 ± 1,723	793 ± 74
	Tonsils	733 ± 43	1,603 ± 249	11,400 ± 2,881	1,166 ± 627
*C2*	LN	45 ± 11	2,246 ± 563	16,072 ± 1,410	5,387 ± 371
	Tonsils	86 ± 93	2,780 ± 917	16,483 ± 3,485	3,486 ± 966
*C3*	LN	52 ± 19	360 ± 30	31,604 ± 4,260	376 ± 77
	Tonsils	60 ± 55	357 ± 66	35,257 ± 15,129	1,101 ± 765
*CD93*	LN	95 ± 30	4 ± 3	6,522 ± 3,428	33 ± 15
	Tonsils	117 ± 83	12 ± 8	6,105 ± 3,210	48 ± 36
*CMKLR1*	LN	19 ± 2	203 ± 107	4,218 ± 698	933 ± 337
	Tonsils	23 ± 16	228 ± 59	4,601 ± 855	466 ± 137

a *Mean number of reads ± SD*.

Finally, Table 5 displays genes that were expressed specifically in the CD14^high^ subset, including *CD68, SLC11A1, MAFB, TFEC, FCGR1A* (CD64), *SIGLEC1* (CD169), *ZFP36L1*, and *CHIT1*. Transcription of these genes confirmed the monocytic lineage identity of the CD14^high^ subset. The latter two genes are associated with monocyte/macrophage development and monocyte-to-macrophage differentiation, respectively. Altogether, these data demonstrate that the gating strategy presented in Figure 4 defines distinct populations within lymph nodes and tonsils that can be attributed to cDC1, cDC2, pDC and monocytic cells.

**Table 5:** Differentially regulated genes in CD14^high^ cells.

**Gene**		**cDC1**	**cDC2**	**pDC**	**CD14^**high**^**
*NFIL3*	LN	384 ± 84^a^	812 ± 43	102 ± 19	9,396 ± 857
	Tonsils	1,008 ± 382	1,887 ± 824	312 ± 67	29,942 ± 11,189
*MAFB*	LN	76 ± 4	3,267 ± 1,406	63 ± 27	28,837 ± 7,820
	Tonsils	251 ± 174	2981 ± 182	123 ± 38	57,937 ± 18,638
*FCGR1A*	LN	139 ± 11	139 ± 27	153 ± 45	578 ± 159
(CD64)	Tonsils	92 ± 53	121 ± 36	130 ± 106	298 ± 52
*CD68*	LN	760 ± 304	854 ± 96	104 ± 23	9,698 ± 3,643
	Tonsils	789 ± 174	1,404 ± 138	146 ± 43	9,759 ± 3,182
*CD163*	LN	4 ± 3	188 ± 122	4 ± 1	19,923 ± 4,775
	Tonsils	211 ± 299	3,076 ± 1,122	99 ± 140	37,033 ± 6,409
*SIGLEC1*	LN	0	11 ± 2	1 ± 1	2,194 ± 792
(CD169)	Tonsils	0	19 ± 16	18 ± 10	545 ± 362
*CCR1*	LN	51 ± 39	1,746 ± 568	15 ± 9	7,810 ± 593
	Tonsils	87 ± 99	1,929 ± 596	35 ± 44	27,185 ± 6,502
*MMP9*	LN	40 ± 35	51 ± 63	1 ± 1	272 ± 158
	Tonsils	134 ± 150	432 ± 329	54 ± 60	6,452 ± 2,785
*TFEC*	LN	56 ± 12	368 ± 114	378 ± 224	2,963 ± 936
	Tonsils	56 ± 35	1,025 ± 191	271 ± 203	5,038 ± 1,639
*SLC11A1*	LN	8 ± 4	165 ± 53	11 ± 14	3,930 ± 1,805
	Tonsils	39 ± 55	132 ± 53	7 ± 9	12,827 ± 3,665
*ZFP36L1*	LN	1,661 ± 169	2,194 ± 353	836 ± 118	4,956 ± 1,225
	Tonsils	2,014 ± 185	2,321 ± 516	1190 ± 125	4,398 ± 1,543
*CHIT1*	LN	0	0	0	1,460 ± 290
	Tonsils	0	0	0	2,414 ± 656

a * Mean number of reads ± SD*.

Moreover, the cell subsets isolated from tonsils and lymph nodes displayed similar PRR expression to their previously described counterparts in porcine blood, with cDC2 and the CD14^high^ subset expressing *TLR2, TLR4*, and *NOD1*, and both cDC subsets and the CD14^high^ subset expressing *NLRP3, CASP1*, and *TLR8* (Supplementary Figures 3C,D). The expression of *TLR3* was restricted to the pDC subset, as in peripheral blood. This subset also displayed the highest expression of *TLR9* and *TMEM173* (coding for the cytoplasmic DNA sensor STING). The RLR genes *DDX58, IFIH1* and *DHX58* were expressed by all four mononuclear-phagocyte populations with the CD14^high^ subset expressing the highest levels. The macrophage-specific C-type lectin receptor *CLEC4E* (MINCLE) was dominantly expressed by the CD14^high^ subset.

### Transcriptional Response of DC in LN Following Virus Infection

Following the identification and characterization of porcine DC subsets in lymphoid tissue at homeostasis, our aim was to determine their specific early transcriptional response to an acute viral infection *in vivo*. To this end, we used CSFV infection as a model for an acute systemic virus infection associated with strong innate immune activation, which has been proposed to be a major factor contributing to immunopathology of the disease (9). The planning of the study was based on the hypothesis that CSFV would enter the host through the tonsils after oronasal infection, likely representing a natural route of infection (38). We reasoned that the virus should have reached the draining lymph nodes 1 day later. Being interested in early time points, we selected 18 and 42 h post infection (p.i.) for collection of tonsils and lymph nodes, respectively. Both of these time points are before the onset of viremia, which is 3 days post infection with highly virulent strains such as Eystrup (39) or Brescia (40). With the PdR strain, viremia is delayed and is detected only at low levels (8). The time points were also selected based on the pathogenesis of virulent CSFV, which was shown to induce lymphoid depletion at 1–2 days post infection (40, 41).

**Figure 6:**
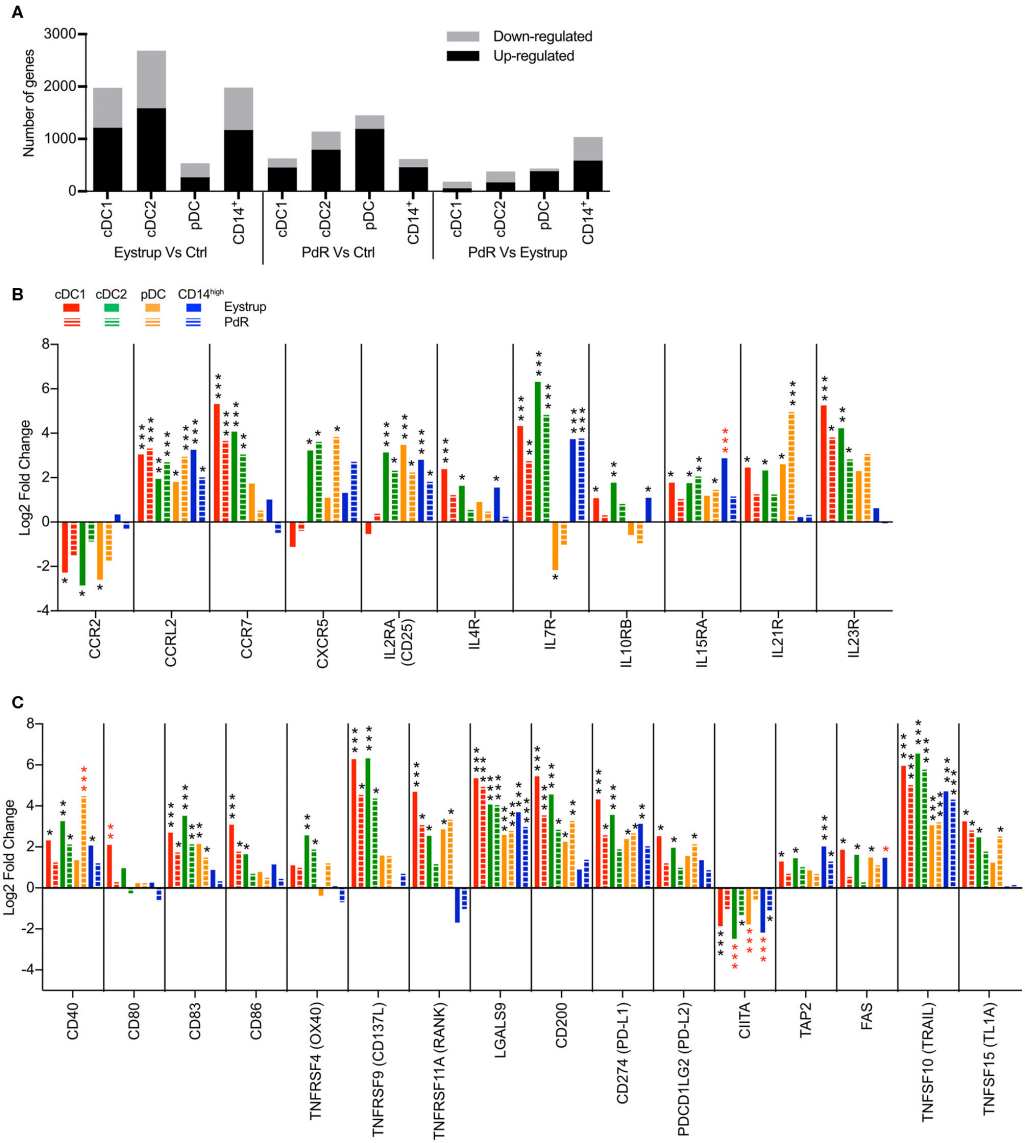
Transcription profiles of LN mononuclear phagocytes after infection with a highly virulent (Eystrup) or a low virulent (PdR) strain of CSFV. cDC1, cDC2, pDC, and CD14^high^ populations were isolated from mandibular and retropharyngeal LN of animals either infected with Eystrup, PdR or left uninfected at 42 h p.i. and their transcriptome was sequenced by RNA-Seq. **(A)** Number of significantly regulated genes (padj < 0.05 in DESeq2 analysis) when comparing cell subsets from Eystrup-infected animals and PdR-infected animals to their counterparts from uninfected animals or comparing subsets between Eystrup- and PdR-infected animals. **(B)** Significantly modulated expression of chemokine and cytokine receptor genes displayed as log2 fold change between Eystrup-infected (solid columns) or PdR-infected (dashed columns) and control animals. **(C)** Significantly modulated expression of genes related to antigen presentation and apoptosis. DESeq2 analysis * padj < 0.05, ** padj < 0.001, *** padj < 0.0001. Red-colored asterisks indicate statistical significance (Padj < 0.05) between Eystrup- and PdR-infected animals for the same cell subset.

Pigs were infected with a highly virulent strain of CSFV responsible for acute disease [vEy-37 derived from the Eystrup strain (7)] or a low-virulent strain leading to chronic infection (Pinar del Rio, PdR) to understand how DC might contribute to the different disease outcomes. The cDC1, cDC2, pDC, and CD14^high^ subsets were sorted from the tonsils of infected animals at 18 h p.i. or from the draining mandibular and retropharyngeal lymph nodes at 42 h p.i. At these very early time points, no clinical signs and no viremia were detected in the animals. High-throughput sequencing was then performed on RNA isolated from sorted cells and the transcriptional profile of each subset was compared between infected and control animals. Very few genes were significantly regulated in the subsets isolated from the tonsils at 18 h post infection. With the PdR strain for example, the expression of 14 genes for pDC, 11 genes for cDC1, and 1 gene for cDC2 changed significantly (padj < 0.05). In contrast, at 42 h p.i. with the highly virulent Eystrup strain, a high number of genes were differentially expressed in cDC subsets and CD14^high^ cells isolated from the LN (1976 genes for cDC1, 2685 genes for cDC2 and 1980 genes for CD14^high^ cells), while pDC displayed fewer regulated genes (537) (Figure 6A). At 42 h p.i. with the low-virulent PdR strain, more genes were differentially regulated in pDC (1451 genes), but fewer in the other subsets (623 for cDC1, 1141 for cDC2 and 617 for CD14^high^).

Figure 6B demonstrates the characteristic chemokine receptor switch in activated DC for both cDC subsets in LN of Eystrup-infected animals, with a downregulation of *CCR2* and an upregulation of *CCR7*. This *CCR7* upregulation was also found in cDC1 and cDC2 subsets of PdR-infected animals. Conventional DC type 2 from all infected animals and pDC from PdR-infected animals displayed an upregulation of *CXCR5*, suggesting an ability to migrate to the parafollicular regions of the lymph nodes.

The cDC2 and pDC subsets also increased their expression of *IL2RA* (CD25), which is involved in the interaction with T cells. We found an increased expression of *IL7R, IL23R* (both strains) and *IL21R* (Eystrup only) by cDC1 and cDC2 subsets, while pDC downregulated *IL7R* (Eystrup) and upregulated *IL21R* following infection (both CSFV strains; Figure 6B).

The activation of the cDC1 and cDC2 subsets was also confirmed by the upregulation of costimulatory molecules *CD40, CD83, CD86, TNFRSF9* (CD137L) and *TNFSRF11A* (RANK), with the exception of *CD40* for cDC1, and *CD86* and *TNFRSF11A* for cDC2 in PdR-infected animals (Figure 6C). Interestingly, some costimulatory molecules were regulated in a more subset-specific manner. Expression of *CD80* was increased only in cDC1 from Eystrup-infected animals (also significant against expression by cDC1 from PdR-infected animals), and *TNFRSF4* (OX40) was upregulated only by cDC2 with both strains (Figure 6C). Plasmacytoid DC displayed an increase in *CD83, TNFRSF11A* and *CD40*, the latter only in PdR-infected animals. At the same time, DC subsets also increased their expression of co-inhibitory molecules such as *CD200* and *CD274* (PD-L1). However, this upregulation of co-inhibitory molecules may not be a specific effect of CSFV infection, as it was also observed for all DC subsets following *in vitro* TLR stimulation.

All subsets isolated from Eystrup-infected animals also showed a decrease in the expression of class II transactivator *CIITA* (Figure 6C). The protein encoded by this gene negatively regulates MHC class II expression following activation of DC (42), a mechanism that would prevent processing and presentation of newly acquired antigens during and following migration from the site of infection to the lymph nodes.

Expression of the death receptor *FAS* was exclusively induced in all subsets isolated from Eystrup-infected animals, whereas *TNFSF10* (TRAIL) was induced in all subsets irrespective of the virus strain.

**Figure 7:**
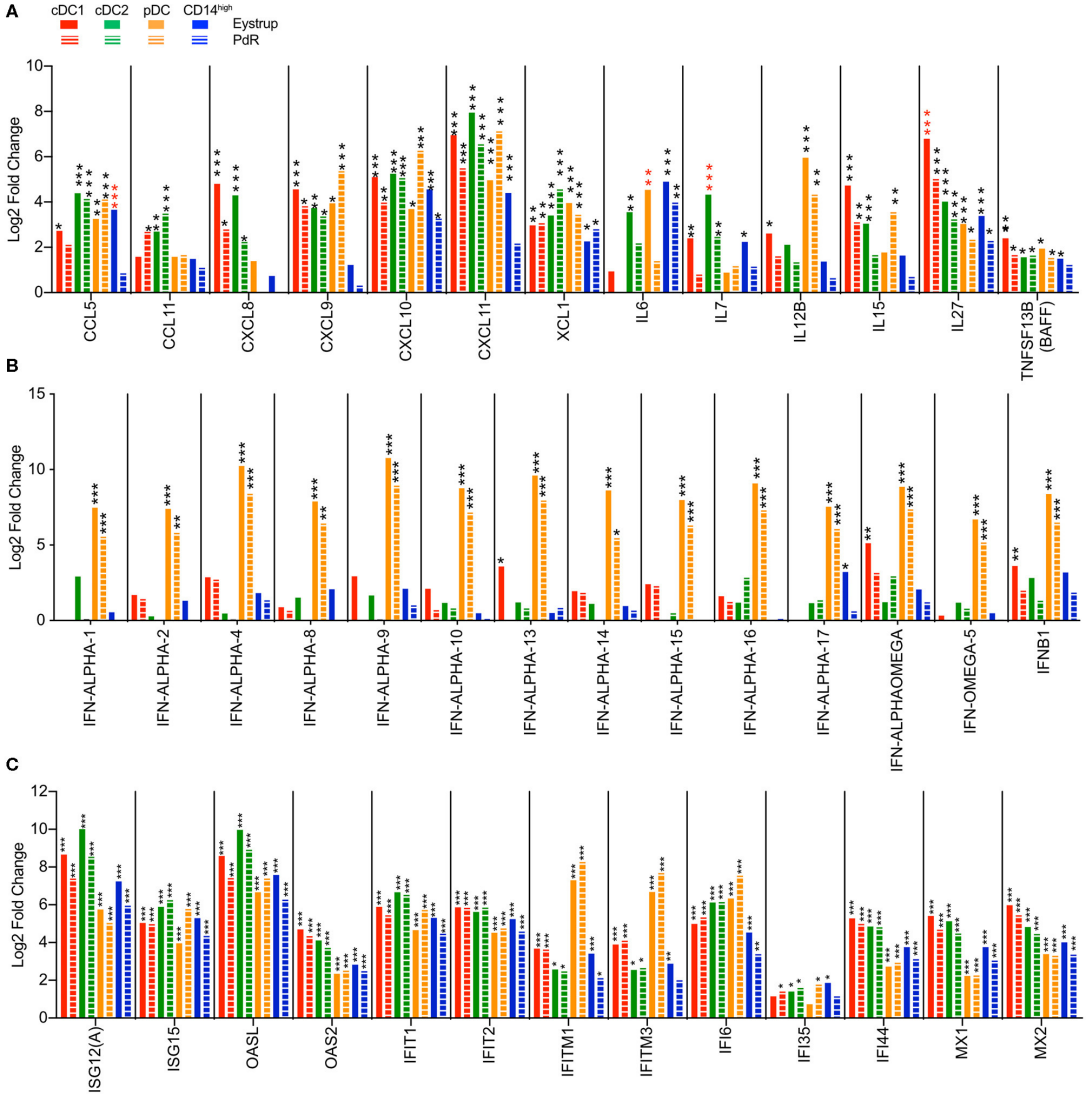
Transcription profiles of LN mononuclear phagocytes after infection with the highly virulent (Eystrup) or the low virulent (PdR) strain of CSFV. cDC1, cDC2, pDC, and CD14^high^ populations were isolated from mandibular and retropharyngeal LN of animals infected with either Eystrup, PdR or left uninfected at 42 h p.i. and their transcriptome was sequenced by RNA-Seq. Expression is displayed as log2-fold change between Eystrup-infected (solid columns) or PdR-infected (dashed columns) and control animals. **(A)** Significantly modulated expression of chemokine and cytokine genes. **(B)** Significantly modulated expression of type I IFN genes. **(C)** Expression of selected interferon-stimulated genes. DESeq2 analysis * padj < 0.05, ** padj < 0.001, *** padj < 0.0001. red-colored asterisks indicate statistical significance (padj < 0.05) between Eystrup- and PdR-infected animals for the same subset.

In both infected groups, all three DC subsets strongly upregulated lymphocyte-recruiting chemokines including *CXCL9, CXCL10*, and *CXCL11* (Figure 7A). However, cDC1 and cDC2 were the only subsets to significantly upregulate the neutrophil attracting *CXCL8* and the eosinophil attracting *CCL11* chemokine genes. Interestingly, the virus infection also promoted the expression of *XCL1*, a chemokine specifically attracting cDC1 (2).

Similar to our *in vitro* results, pDC and cDC1 were the only subsets significantly upregulating *IL12B*. However, while pDC produced high levels of this mRNA after infection with both CSFV strains, cDC1 seemed to respond only to the highly virulent Eystrup strain (Figure 7A). The gene encoding T-cell homeostatic cytokine IL-7 (*IL7*) was induced in cDC1, cDC2, and monocytic cells after infection with Eystrup, but only in cDC2 with PdR. The gene encoding IL-27 *(IL27)*, a cytokine regulating T-cell responses, was induced in all subsets, irrespective of the CSFV strain. Gene transcription for the B-cell activating cytokine BAFF (*TNFSF13B*) was also increased in all subsets (Figure 7A).

As expected, pDC were clearly the main—and in most cases the only—subset upregulating type I interferon genes (Figure 7B). Although higher mean counts for all IFN type I genes were found with Eystrup when compared to PdR, this difference was not statistically significant at the single-gene level (Figure 7B). Conventional DC type 1 isolated from animals infected with the Eystrup strain also significantly upregulated *IFN-ALPHA-13, IFN-ALPHAOMEGA*, and *IFNB1*. Type I IFN expression was associated with the upregulation of many interferon-stimulated genes (ISG) by all subsets (Figure 7C), among them the anti-viral genes *OASL, OAS2, IFIT1, IFIT2, MX1*, and *MX2*. This strong induction of IFN-responsive genes also included certain PRR genes known to be responsive to IFN, including *DHX58* (LGP2), *IFIH1* (MDA5) and *DDX58* (RIG-I) (Supplementary Figure 4A). Interestingly, *DDX60*, encoding a positive regulator of RIG-I- and MDA5-dependent IFN type I responses (43), was also highly upregulated by the two viruses (Supplementary Figure 4A).

As visible in Supplementary Figure 4B, cDC1 and cDC2 were the only subsets to show an increase in expression of genes related to the NFκB pathway such as *NFKB1* (only Eystrup for cDC1 and cDC2), *NFKB2* (only Eystrup for cDC2), *REL* (only Eystrup for cDC1 and cDC2) and *RELB* (only Eystrup for cDC2). Genes related to the JAK/STAT pathway were also upregulated in cDC1 (*JAK1* and *JAK2* in the Eystrup group) and cDC2 (*JAK1* in the Eystrup group) and in all subsets for the different STAT genes (Supplementary Figure 4B). Genes related to TLR signaling were significantly regulated with an increase in *IRAK2* and *IRAK4* expression in the monocytic cell subset from the Eystrup group, while *IRAK1* was downregulated in cDC2 from both infected groups and in pDC from the PdR group (Supplementary Figure 4C). Both cDC subsets also upregulated *TRAF1, TRAF2* (only in Eystrup animals) and *TRAF4* (except for cDC1 from PdR-infected animals). Transcription of *TRAF6* was found to be exclusively upregulated in cDC2 from Eystrup-infected animals.

### Modular Analyses of DC Responses After Virus Infection

**Figure 8:**
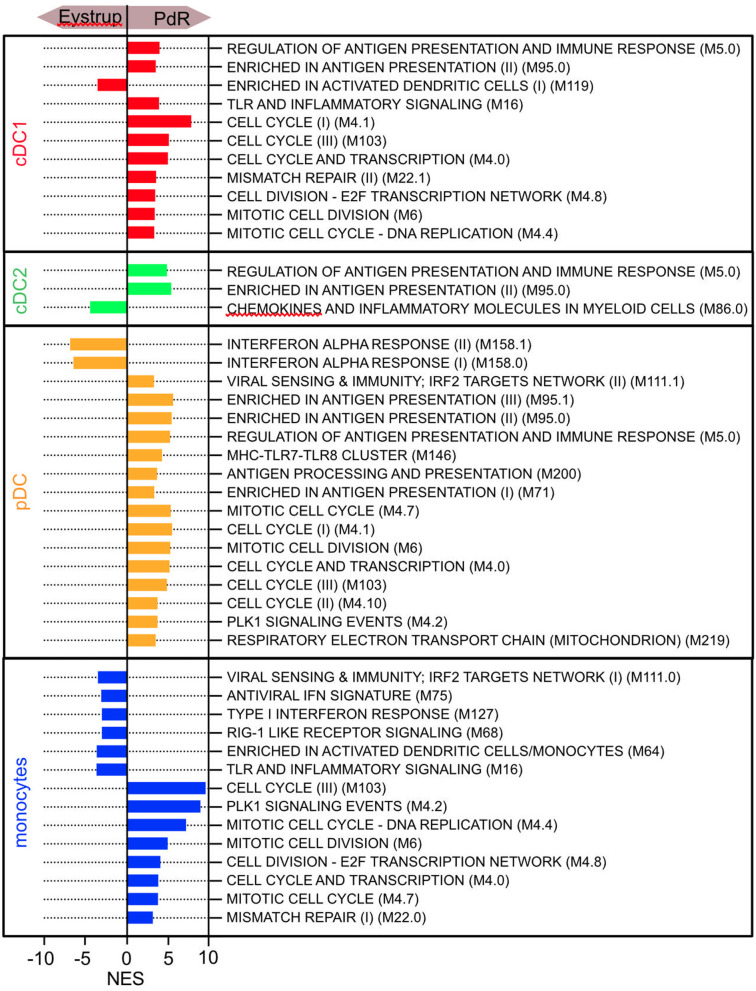
Gene-set enrichment analysis of sorted DC and monocytic cells from lymph nodes using 86 selected gene sets that were derived from the BTM described by Li et al. (25). The normalized enrichment scores (NES) for all modules with a FDR < 0.05 are shown.

To further investigate possible differences in DC/monocytic cell responses between the two strains of CSFV, we applied GSEA to the data set of the sorted LN DC using 86 selected gene sets that were derived from the blood transcription modules (BTM) described by Li et al. (25) and modified for pigs by Matthijs et al. (26). The expression of modules containing IFN-α/β genes was clearly higher in pDC of the Eystrup-infected animals (Figure 8). Along the same line, we found higher scores for modules containing interferon responsive genes in the monocytic cells of Eystrup-infected animals. In contrast, we found a significant enrichment of some modules related to antigen presentation such as M5.0 and M95.0 in all DC subsets from the PdR-infected pigs when compared to the Eystrup group. Furthermore, many modules associated with cell cycle were also enriched in cDC1, pDC and monocytes derived from PdR-infected animals. In conclusion, these analyses revealed CSFV-strain-dependent differences in the activation of antigen presenting cells in the LN.

## Discussion

With the unique role of DC at the interface of innate and adaptive immunity and their ability to induce and regulate adaptive immune responses, the precise mapping of DC responses to immunostimulants or infection is of importance for the development of improved vaccines and novel immunotherapeutic strategies. To rationally and efficiently achieve this for the pig, the identification and characterization of porcine DC and monocytic cell subsets and their innate response profiles is crucial. We utilized two complementary ways of studying DC subset responses: (i) direct *in vitro* stimulation of sorted porcine blood DC subsets with defined TLR ligands to assess the stand-alone transcriptional response of each subset, and (ii) the transcriptional response of sorted subsets isolated from lymphoid tissue early after *in vivo* viral infection.

First of all, we compared the response of DC subsets and monocytes to the TLR1/2 ligand PAM3Cys which is able to activate both porcine DC and monocytes (3). When considering the number of differentially expressed genes, cDC1 and cDC2 were highly responsive to this ligand, while monocytes, despite the highest expression of *TLR1* and *TLR2*, had the lowest response. All DC subsets displayed an activated profile with downregulation of *CCR2*, and upregulation of *CCR7* and costimulatory molecules, while only cDC1 and cDC2 subsets upregulated the expression of a broader range of chemokine receptors targeting both T cells and innate immune cells. Despite low expression levels of *TLR2*, pDC were still responsive to PAM3Cys, but the number of significant differentially regulated genes was lower than following TLR3, TLR7, or TLR9 stimulation, the three most highly expressed TLRs in porcine pDC. The present work also confirms the previous observation (3) that porcine blood pDC are an important source of TNF following TLR stimulation, and also upregulate a broad range of type I IFN as well as both *IL12A* and *IL12B* following CpG ODN stimulation. Furthermore, pDC were shown to upregulate transcription of *XCL1*, encoding a chemokine that attracts cDC1. Altogether, this supports the idea that pDC have a central role for Th1 responses in the pig.

In a parallel approach, we compared the effects of TLR1/2, TLR3, TLR7, TLR7/8, and TLR9 ligands on pDC with the aim to determine ligand-specific transcriptional signatures and the most potent ligand for porcine pDC. While all ligands induced a switch in chemokine receptors required for migration to lymphoid tissue, and induced co-stimulatory molecules, only CpG ODN appeared to induce a “full-blown” IFN type I response, as well as the transcription of *IL12* genes. This indicates that CpG ODN would be a particularly suitable ligand to target porcine pDC for induction of potent antiviral and Th1 responses. Furthermore, resiquimod was found to be very potent in the upregulation of inflammatory cytokines and chemokines as well as in the induction of T-cell recruiting chemokines.

In order to study responses of porcine DC in lymphoid tissues, our next aim was to establish a suitable antibody staining panel for DC populations in tonsils and lymph nodes. After extensive phenotyping, we found that both in blood and in lymphoid tissue cDC1 can be defined as CD14^−^CD172a^−/low^CADM1^+^, cDC2 as CD14^−^CD172a^+^CADM1^+^ and pDC as CD14^−^CD172a^+^CADM1^−^CD4^+^. In lymphoid tissue, cDC1 expressed the highest levels of CD205 and wCD11R1, cDC2 expressed CD1 and low levels of CSF1R, and pDC expressed the IL-3 receptor CD123. The identification of these subsets was confirmed by transcriptomic analyses. As in the blood, only DC subsets expressed the cross-species pan-DC marker *FLT3*. DC subset-specific markers found across species include *BATF3, XCR1, ANPEP* (CD13), *DPP4* (CD26) for cDC1, *FCER1A* for cDC2, and *TCF4* (E2-2) and *RUNX2* for pDC. We also found that the expression of *IRF4* and *IRF8* in porcine DC subsets isolated from lymph nodes and tonsils matched the pattern found in porcine blood DC and in DC of other mammalian species (44–47), with pDC expressing the highest levels of both, cDC1 expressing more *IRF8* than cDC2, and cDC2 expressing more *IRF4* than cDC1. Another study described DC subsets in the porcine tonsil using a different gating strategy, involving also class II MHC expression, and found similar subset-specific transcription patterns of *FLT3, TCF4, XCR1*, and *CSF1R*, with the noticeable exception of *IRF4* which was not found to be expressed in porcine pDC isolated from tonsils (48). The transcription of TLR genes in porcine DC from lymphoid tissue was also found to be comparable to the transcription in porcine DC subsets from blood (3), in particular the pDC-restricted expression of *TLR3*. Another peculiarity of porcine pDC was found to be shared between blood and lymphoid tissue: the high expression of complement-related genes such as *C2, C3*, or *CD93*. Interestingly, CD93 was shown to be involved in delivery of CpG ODN to endosomal TLR9 (49) and could be an interesting way of targeting and activating porcine pDC. Both porcine cDC subsets from lymphoid tissues also showed expression of markers that were not expressed on their blood counterparts. As observed in mouse (32, 45), porcine cDC1 from tonsils and lymph nodes expressed the gene for CD103 (*ITGAE*), a specific phenotypic feature of migratory and tissue cDC1. Porcine lymphoid tissue cDC2 expressed high levels of *FSCN1*, a gene associated with DC migration to lymph nodes (35), and *PLA2G2D*, a gene expressed by DC in lymphoid organs (36). Furthermore, similar to what has been described in porcine lungs (50) and in human tonsils and lymph nodes (51, 52), porcine lymphoid tissue cDC2 expressed *CD207*.

The present study also identified a monocytic cell subset in porcine lymph nodes and tonsils characterized by high levels of CD14 expression, which clustered with monocytes and monocyte-derived macrophages in PCA, and expressed the monocyte/macrophage-specific lineage markers *CD64* (53), *CD68* (54) and *SLC11A1* (55). The expression of *ZFP36L1*, which is selectively upregulated during monocyte/macrophage differentiation (56), and the macrophage-specific transcription factors *TFEC* (57) and *MAFB* (58), together with *CHIT1*, a gene involved in monocyte-to-macrophage differentiation (59), would indicate that this CD14^high^ population corresponds to monocyte-derived macrophages.

This precise and unambiguous identification of the DC subsets in lymphoid tissue allowed us to address the final aim, which was the early DC response following CSFV infection. Based on published work (10), we isolated DC subsets from tonsils at 18 h p.i. However, we could not observe any differences in gene expression when comparing uninfected with CSFV-infected animals, suggesting that tonsils might have been harvested too early following infection. However, we observed many transcriptional changes in DC subsets from mandibular and retropharyngeal lymph nodes at 42 h p.i. At this later time point, both cDC subsets displayed more changes in gene transcription following infection with the highly virulent Eystrup strain than with the low-virulent PdR strain. Nevertheless, the observed expression profiles of immune-related genes were overall very similar between both CSFV strains. Interestingly, pDC isolated from PdR-infected animals displayed more differentially regulated genes than pDC isolated from Eystrup-infected animals. The observed differences might reflect exhaustion of pDC during infection with the highly virulent strain. On the other hand, it should be pointed out that the PdR strain has a unique poly-uridine sequence of the 3′UTR, which possibly promotes pDC activation (8, 60).

An interesting observation was that co-stimulatory molecules targeting resting T cells, including CD80 and CD86, were not markedly induced, while inhibitory/regulatory receptors including *LGALS9* (Galectin-9), *CD200* and *CD274* (PD-L1) were strongly induced in all DC subsets. Although this upregulation was also observed following TLR ligand stimulation *in vitro*, future studies are required to address if this DC activation profile could be responsible for the early defects in adaptive immune responses that are typical for CSFV (9). As previously observed in the blood of CSFV-infected pigs (61), we observed a prominent increase in the gene expression of the apoptosis-inducing death receptor TRAIL by all DC subsets, which, together with the strong expression of T-cell attracting chemokines, could contribute to the lymphopenia observed following CSFV infection. For both the inhibitory and the death receptors, we often observed higher levels with the virulent strain of CSFV, which would support a possible pathogenic role of these responses.

Very high serum levels of type I IFNs are characteristic for the acute phase of CSF. Our study indeed supports a central role of pDC in this response, as demonstrated by the upregulation of many type I IFN genes in pDC from both, Eystrup and PdR-infected animals. Previous work has shown that less virulent strains of CSFV resulted in less IFN-α in the serum of infected animals compared to highly virulent strains (12, 61, 62). Although we did not observe significant differences in type I IFN expression at the level of individual IFN type I genes, GSEA analyses with modules composed of these genes demonstrated higher IFN responses in pDC from Eystrup-infected animals. These gene-set enrichment analyses also demonstrated that modules related to antigen presentation and cell cycle were more strongly upregulated following infection with the PdR strain when compared to the Eystrup strain. For future investigations, we therefore propose to address the general role of a proliferative DC response during an acute virus infection, as well as the question if the observed differences in DC activation could be partly responsible for the differences in pathogenicity between the two CSFV strains.

The *in vivo* data is also relevant to understand the functional specialization of DC subsets in the pig. In addition to the unique ability to express IFN type I genes, *IL12B* transcription was upregulated by pDC from all infected animals and by cDC1 from Eystrup-infected animals, confirming our *in vitro* data and identifying porcine pDC as an important source of this Th1-promoting cytokine. Another indication for subset-specific functions is the observation that *CXCR5* expression was induced in cDC2 but not in cDC1, which could reflect the increased ability of cDC2 to migrate to the parafollicular regions of the lymph node.

From a methodological point of view, it should be mentioned that *in vitro* stimulation of sorted subsets can give valuable information on how defined subsets can be directly activated in an isolated context, and is suitable to screen for immunostimulants. Nevertheless, the data obtained cannot fully reflect the more complex *in vivo* situation in which DC activation is influenced by cell interactions and soluble factors in tissues and in the local immunological environment. However, during an *in vivo* infection, the timing for the interaction of the stimulus or pathogen with the immune cell is difficult to control. Indeed, we observed some differences in innate responses of DC subsets *in vivo*. For example, *CCR7* upregulation was restricted to cDC in the lymph nodes after *in vivo* infection, but was observed *in vitro* in blood pDC irrespective of the TLR ligand used for stimulation. The lack of *in vivo* activated CCR7^+^ pDC suggests that—at this stage of infection—only cDC migrate from infection sites to the lymph nodes. Type and cellular origin of expressed chemokines also differed when *in vivo* and *in vitro* data was compared, again highlighting the multitude of factors influencing DC activation *in vivo*.

Taken together, the data presented here will help to understand the response of antigen-presenting cells during infection and will help to target DC for the development of efficient immunotherapeutic interventions and vaccine strategies.

## Data Availability Statement

The datasets presented in this study can be found in online repositories. The names of the repository/repositories and accession number(s) can be found below: https://www.ebi.ac.uk/ena, PRJEB37564, PRJEB37565.

## Ethics Statement

The animal studies were reviewed and approved by the Amt für Landwirtschaft und Natur LANAT, Veterinärdienst VeD, Bern, Switzerland (licenses BE88/14, BE131/17, BE105/15).

## Author Contributions

GA performed laboratory work, analyzed the data, and wrote the manuscript. MG, SP, and ML performed laboratory work and analyzed data. IK, ST, and RB performed bioinformatic analyses. LG provided essential analyses. AS and NR wrote the manuscript, designed, and supervised the overall project. All authors contributed to the article and approved the submitted version.

## Conflict of Interest

The authors declare that the research was conducted in the absence of any commercial or financial relationships that could be construed as a potential conflict of interest.
