# Supplementary material for: High-Resolution Profiling of Innate Immune Responses by Porcine Dendritic Cell Subsets in vitro and in vivo
Source: Front Immunol. 2020 Jul 7;11:1429. doi: 10.3389/fimmu.2020.01429 (PMC7358342; doi:10.3389/fimmu.2020.01429)
Supplement: Supplementary file 1 [file Data_Sheet_1.PDF]

Supplementary data

Transcriptomic profiling of innate immune responses by porcine dendritic cell subsets *in vitro and in vivo*

Gael Auray[1,2], Stephanie C. Talker [1,2], Irene Keller[3], Sylvie Python[1], Markus Gerber[1], Matthias Liniger[1,2], Llilianne Ganges[4], Remy Bruggmann[5], Nicolas Ruggli[1,2], and Artur Summerfield[1,2]

[1]Institute for Virology and Immunology, 3147 Mittelhäusern, Switzerland

[2]Department of Infectious Diseases and Pathobiology, University of Bern, 3012 Bern, Switzerland.

[3]Department for Biomedical Research and Swiss Institute of Bioinformatics, University of Bern, 3012 Bern, Switzerland

[4]OIE Reference Laboratory for classical swine fever, IRTA-CReSA, Barcelona, Spain

[5]Interfaculty Bioinformatics Unit and Swiss Institute of Bioinformatics, University of Bern, 3012 Bern, Switzerland.

Corresponding author:

Artur Summerfield,

Institute for Virology and Immunology,

Sensemattstrasse 293

Email: artur.summerfield@ivi.admin.ch

CH-3147 Mittelhäusern, Switzerland

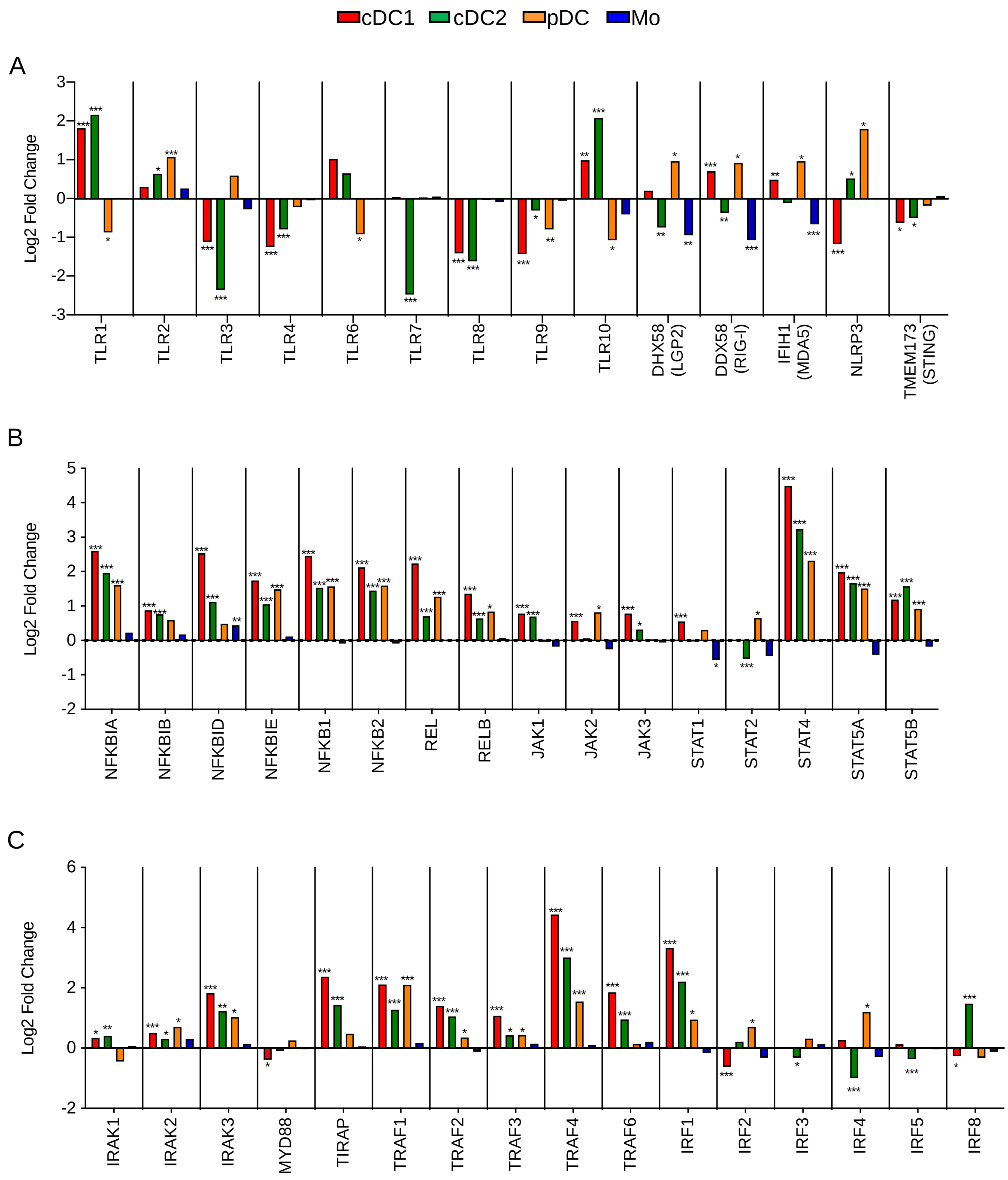

Supplementary Figure 1. (A) Changes in expression of PRR genes and genes related to signaling pathways in porcine blood mononuclear phagocytes following PAM3Cys stimulation. cDC1, cDC2, pDCs and monocytes from 3 different animals were sorted by FACS and stimulated 3h with 10 µg/ml of PAM3Cys or left unstimulated as control. (B) Selected chemokine and cytokine receptor gene expression shown as log2 fold change between PAM3Cys-stimulated cells and their corresponding non-stimulated control. (C) Expression of selected costimulatory molecules shown as log2 fold change between PAM3Cys-stimulated cells and their corresponding non-stimulated control. DESeq2 analysis, padj: * p<0.05, ** p<0.001, *** p<0.0001.

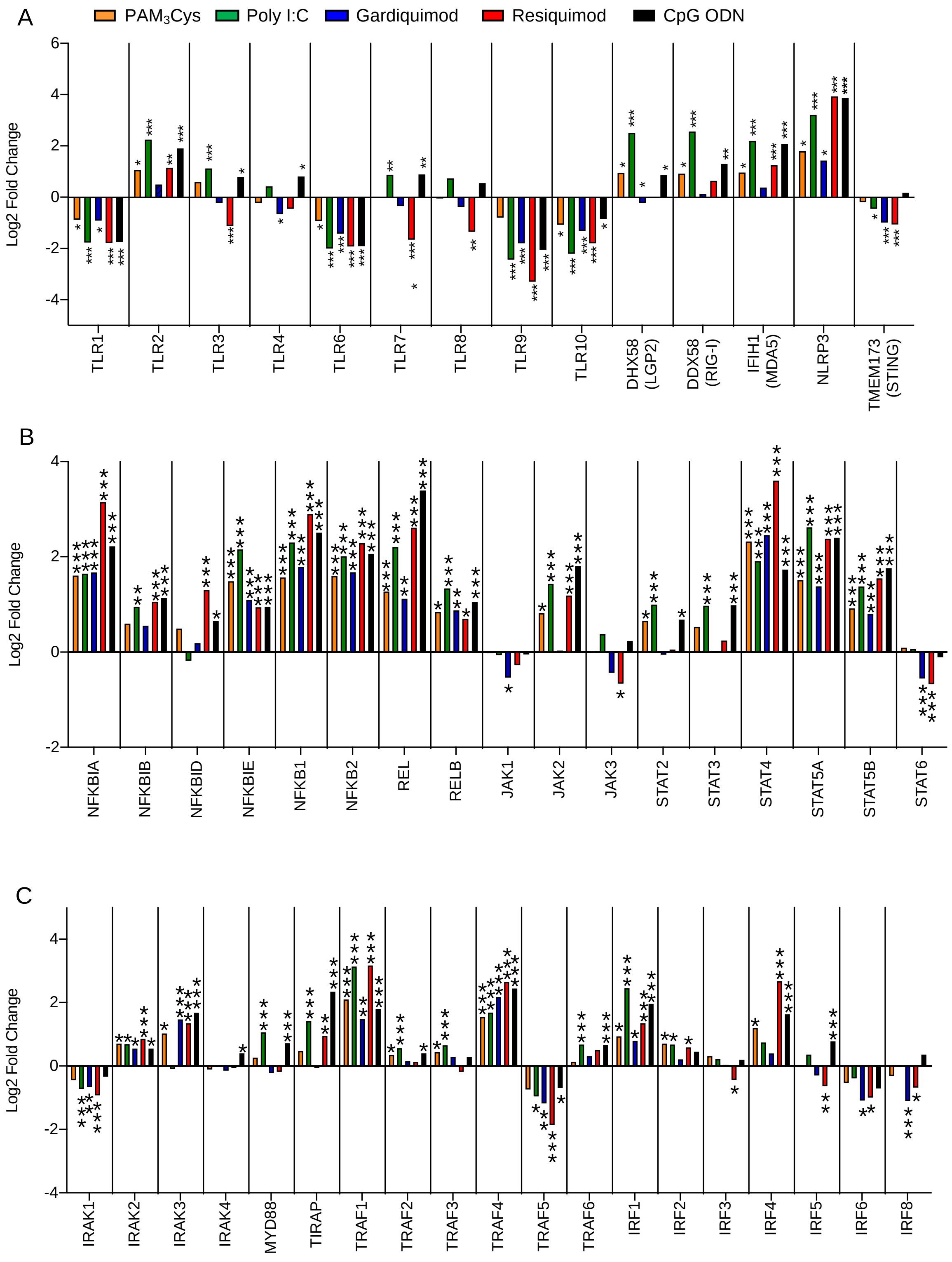

Supplementary Figure 2. Changes in expression of PRR genes (A) and cell signaling genes (B, C) in porcine pDCs following TLR stimulation. pDCs from 3 different animals were sorted by FACS and stimulated with 10 µg/ml of PAM3Cys, 10 µg/ml poly I:C, 5 µg/ml gardiquimod, 5 µg/ml resiquimod, 5 µg/ml CpG ODN D32, or left were unstimulated as control. Selected PRR genes shown as log2 fold change between TLR ligand-stimulated pDCs and control pDCs. DESeq2 analysis, padj: * p<0.05, ** p<0.001, *** p<0.0001.

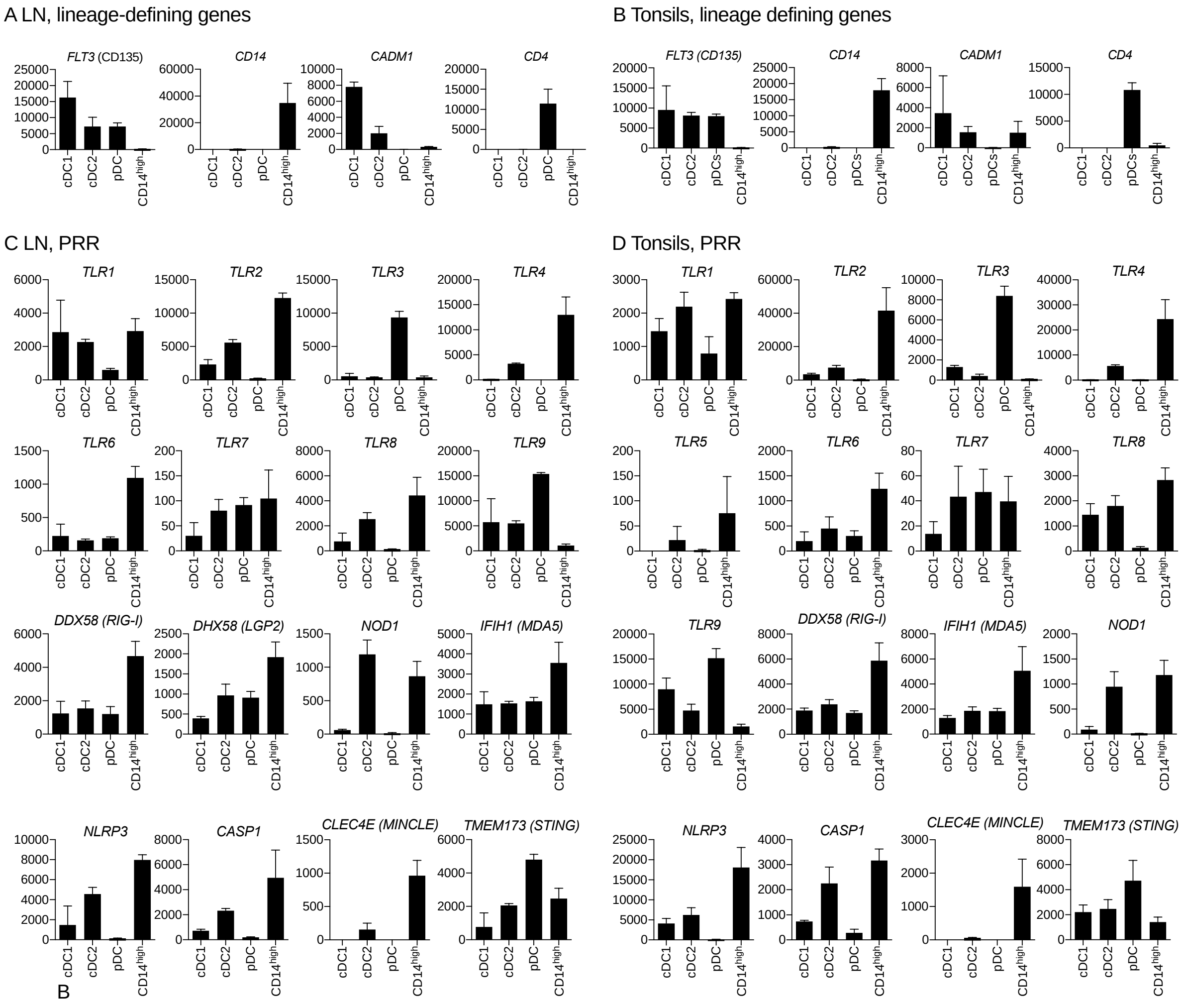

Supplementary Figure 3. A, B. Gene expression of lineage-defining markers used in FCM for LN and tonsils. C, D. PRR gene expression profiles of cell subsets isolated from lymph nodes and tonsils. The data show the mean number of reads and standard deviation (SD) from three different animals.

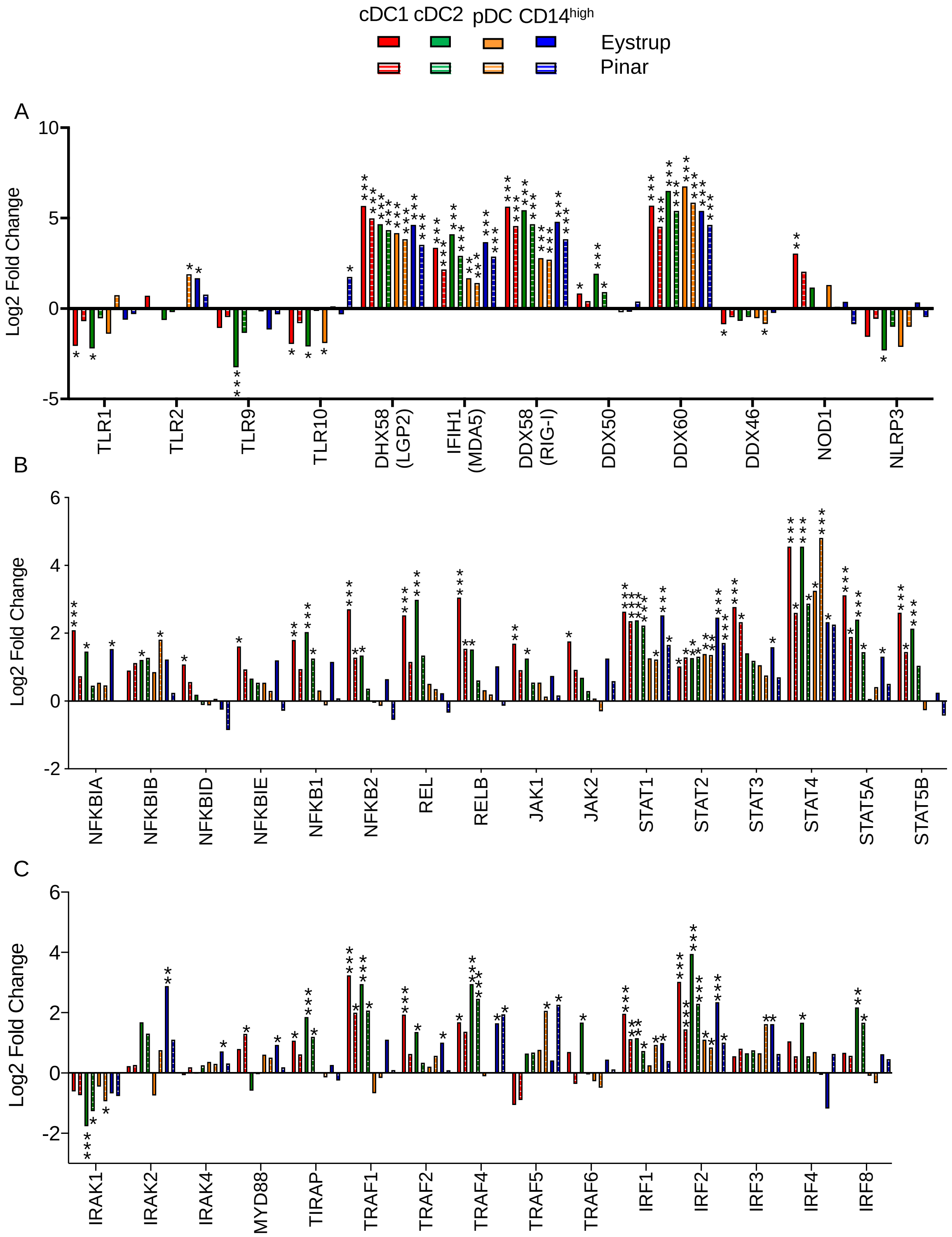

Supplementary Figure 4. Changes in expression of cell signaling genes in LN antigen presenting cells following *in vivo* infection with CSFV. Pigs were infected with either highly virulent CSFV Eystrup or low-virulent PdR. After 42h, DC and monocytic cells were isolated from the LN and analyzed by RNA-Seq. Selected cell signaling genes shown as log2 fold change between TLR ligand-stimulated pDCs and control pDCs. DESeq2 analysis, padj: * $p<0.05$, ** $p<0.001$, *** $p<0.0001$.
